# MMP8/PPAR-γ regulation of macrophage-mediated inflammatory response in the pathogenesis of acute-on-chronic liver failure

**DOI:** 10.1038/s41419-026-08793-z

**Published:** 2026-04-27

**Authors:** Ying Xiao, Luyuan Ma, Xinyang Li, Shilong Dong, Jiachao Wang, Yuexia Liu, Yadong Wang, Caiyan Zhao

**Affiliations:** 1 Department of Infectious Disease, the Hebei Medical University Third Hospital, Shijiazhuang, China; 2 Hebei Clinical Medical Research Center of Infectious Diseases, Shijiazhuang, China; 3 Hebei Key Laboratory for Diagnosis, Treatment, Emergency Prevention and Control of Critical Infectious Diseases, Shijiazhuang, China; 4 Key Laboratory of Immune Mechanism and Intervention on Serious Disease in Hebei Province, Department of Immunology, Hebei Medical University, Shijiazhuang, China

**Keywords:** Diseases, Biomarkers

## Abstract

Acute-on-chronic liver failure (ACLF) is a critical syndrome marked by severe illness, rapid progression, and poor prognosis. We aimed to investigate immunoregulatory mechanisms governing macrophage function during ACLF progression and identify potential molecular therapeutic targets. Differentially expressed genes (DEGs) in macrophages from patients with ACLF were identified using Gene Expression Omnibus datasets and single-cell RNA sequencing. Their expression patterns and prognostic value were assessed in 222 individuals, including healthy controls, patients with chronic hepatitis B, with hepatitis B cirrhosis, and those with hepatitis B virus-related ACLF. In vitro experiments using inhibitors, plasmid transfection, and transcriptome sequencing were performed to clarify how key DEGs regulate macrophage polarization. An ACLF mouse model induced by CCl₄/D-GalN/LPS was used for in vivo validation. Matrix metalloproteinase 8 (MMP8) emerged as a significantly upregulated gene in macrophages during ACLF. MMP8 levels were elevated in liver tissue, serum, peripheral blood mononuclear cells, and CD86⁺ macrophages and showed strong diagnostic efficacy for the early identification and progression prediction of ACLF. Functional studies revealed that MMP8 promotes macrophage M1 polarization, pro-inflammatory cytokine release, and pyroptosis through the peroxisome proliferator-activated receptor gamma (PPAR-γ) pathway. In vivo, the MMP8/PPAR-γ axis amplified hepatic inflammation and liver necrosis. These findings highlight a central role for the MMP8/PPAR-γ pathway in ACLF pathogenesis and highlight its potential as a molecular target for future therapy.

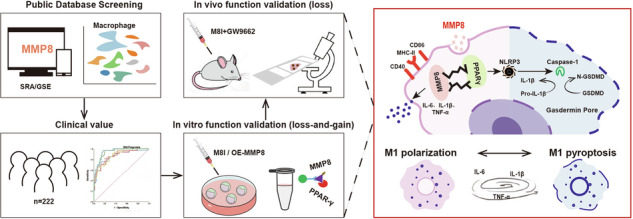

## Introduction

Acute-on-chronic liver failure (ACLF) is a serious form of liver injury triggered by various precipitating factors on the basis of chronic liver disease, leading to significant impairment or decompensation of hepatic synthesis, detoxification, metabolism, and biotransformation. Clinically, it manifests as jaundice, coagulation dysfunction, hepatorenal syndrome, hepatic encephalopathy, and ascites. ACLF is severe, progresses rapidly, and has a poor prognosis, with an incidence of 5.7–20.1 cases per 1000 person-years [1] and a 28- or 90-day mortality rate of 20–80% [2, 3]; hepatitis B virus-related ACLF (HBV-ACLF), even with optimal comprehensive therapy, has a mortality rate as high as 50–90% [4]. However, the mechanisms underlying ACLF exacerbation remain unclear. Although liver transplantation, artificial liver support systems, and other therapeutic approaches benefit some patients, these interventions do not specifically inhibit hepatocyte inflammatory necrosis or enhance liver regeneration. Furthermore, the high cost and limited availability of donor organs restrict treatment options for many patients. Therefore, elucidating the pathogenesis of ACLF at the basic research level, identifying key molecular events and signaling pathways involved in hepatocyte injury, necrosis, and regeneration, and improving early clinical warning and therapeutic strategies to reduce mortality have become urgent and central challenges in hepatology research.

Previous studies have shown that marker genes for innate immune cells, including monocytes and dendritic cells, are significantly enriched in the peripheral blood mononuclear cells (PBMCs) of patients with ACLF, whereas genes associated with adaptive immune cells are significantly reduced, indicating pronounced activation of the innate immune response in ACLF [5]. Patients with ACLF exhibit abnormal host immune reactions and systemic inflammatory dysregulation. The “endotoxin–macrophage activation–cytokine storm” cascade is recognized as a central pathogenic mechanism of ACLF and a major driver of its onset and progression [6]. Advanced techniques such as single-cell sequencing and spatial transcriptomics have revealed multiple macrophage subsets within the liver, collectively regulating immune function, inflammatory status, and hepatic homeostasis. Consequently, macrophage phenotypic changes directly influence disease progression and clinical outcomes [7]. In the early stage of HBV-ACLF, hepatocyte-derived debris promotes polarization of intrahepatic macrophages toward the M1 (pro-inflammatory) phenotype, leading to the release of cytokines and chemokines such as tumor necrosis factor alpha (TNF-α), interleukin-1 (IL-1), IL-6, and C-C motif chemokine ligand 2. These mediators recruit additional immune and inflammatory cells into the liver, further exacerbating hepatic inflammation. As the disease progresses, the number of intrahepatic macrophages decreases rapidly, accompanied by a significant accumulation of blood- or bone marrow-derived M2 (anti-inflammatory) macrophages, which suppress the inflammatory response by secreting anti-inflammatory factors such as IL-10 and transforming growth factor-β (TGF-β) [8, 9]. Pyroptosis is a pro-inflammatory form of cell death characterized by inflammasome activation, cell lysis, and the release of inflammatory mediators, including IL-1β and IL-18, which further recruit inflammatory cells and amplify the immune response [10]. Pyroptosis is closely associated with macrophage polarization and may contribute to excessive inflammation in acute septic liver injury [11]. Thus, macrophage activation states reflect their complex, dual role in ACLF progression. Timely switching of macrophages from the pro-inflammatory M1 phenotype to the anti-inflammatory M2 phenotype is crucial for regulating disease development. Therefore, identifying distinct monocyte and macrophage populations, uncovering novel regulatory molecules, and elucidating their immunoregulatory mechanisms are essential for early disease detection, targeted therapy, and improving patient outcomes. Accordingly, we aimed to investigate the molecular mechanisms by which macrophages contribute to systemic inflammation and organ injury in ACLF, identify key regulatory genes involved in macrophage activation, and explore potential molecular targets for diagnosis, prognosis, and anti-inflammatory therapy.

In this study, by analyzing public transcriptomic and single-cell sequencing datasets for ACLF and validating the findings in clinical samples—including liver tissue, serum, PBMCs, and CD86⁺ macrophages from patients with ACLF—we identified matrix metalloproteinase 8 (MMP8) as a key differentially expressed gene with diagnostic and prognostic significance. Furthermore, in vivo and in vitro experiments demonstrated that MMP8 inhibits peroxisome proliferator-activated receptor gamma (PPAR-γ), promotes macrophage M1 polarization and pyroptosis, and drives systemic inflammation and organ injury in ACLF. These findings provide new insights into the mechanisms underlying systemic inflammation and immune dysregulation in ACLF and offer a theoretical foundation for the development of novel molecularly targeted therapies.

## Methods

### Differential gene screening and processing

ACLF-related datasets were retrieved from the GEO database. Differential expression analysis was conducted with R software [|log₂ (fold change, FC) | ≥ 1 and adjusted *P* < 0.05]. GEO datasets containing patients’ prognosis information were retrieved for external validation. The single-cell RNA sequencing (scRNA-seq) data from the SRA project PRJNA913603 were downloaded and integrated using CellRanger and Seurat, followed by clustering, cell-type annotation, and differential expression analysis (|log FC | > 1 and *P* < 0.05).

### Clinical study and specimen processing

Healthy control (HC), chronic hepatitis B (CHB), hepatitis B cirrhosis (LC), and HBV-ACLF patients were consecutively enrolled between October 2023 and April 2025. Diagnoses of ACLF, LC, and CHB followed the guidelines of the Asian Pacific Association for the Study of the Liver, European Association for the Study of the Liver, and American Association for the Study of Liver Diseases [12–14]. The estimated sample size ratio between the non-ACLF and ACLF groups was set at 1:1. With a two-sided type I error margin of 7% and a target confidence level of 95%, a minimum of 196 patients was required for the study. ACLF was also divided into pre, early, intermediate, and advanced stages according to jaundice and coagulation in ACLF patients [15]. Clinical and laboratory indicators were collected, and patients were followed up for 28/90 days. Peripheral blood was collected to separate serum and PBMCs (#LTS10771, TBD); liver tissue samples were obtained, all properly stored for subsequent analysis. The study protocol was approved by the Medical Ethics Committee of Hebei Medical University Third Hospital (No: K2025-156-1). All the procedures were conducted in accordance with the principles of the Declaration of Helsinki, and written informed consent was obtained from all participants before enrollment.

### Cell culture and in vitro intervention

THP-1 (#CL-0233, Procell), RAW264.7 (Cell Bank, Chinese Academy of Sciences), THLE-2 (#iCell-h388, iCell), and 293 T (#iCell-h477, iCell) cells were cultured in appropriate medium. All cell lines were maintained at 37 °C in a humidified 5% CO₂ incubator. THP-1 cells were differentiated into macrophages using 100 ng/mL phorbol 12-myristate 13-acetate (PMA, #HY-18739; MCE). After 24 hours, the medium was replaced, and cells were stimulated with 100 ng/mL LPS (#HY-D1056, MCE) and 20 ng/mL IFN-γ (#DC054, Novoprotein) for 0, 12, or 24 hours before RNA and protein extraction. RAW264.7 cells were stimulated with 200 ng/mL LPS for 0, 6, or 12 hours prior to harvesting for RNA and protein extraction. Cell viability was detected by CCK-8 assay (#RC3028, ReportBio). MMP8 and PPAR-γ overexpression plasmids were transfected (synthesized by Beijing Bomaide Biotechnology Co., Ltd., China); MMP8 inhibitor (M8I, #236403-25-1, Cayman) was used; and PPAR-γ expression was regulated with 10 μM GW1929 (#HY-15655, MCE) / 10 μM GW9662 (#HY-16578, MCE).

### ACLF mouse model construction and grouping

Forty-five 3–4-week-old male BALB/c mice were purchased from Beijing Huafukang Bioscience Co., Ltd. (Certificate No: SCXK (Jing) 2024-0003) and housed in a standard-specific pathogen-free environment. Temperature, humidity, light/dark cycle, and noise/ammonia concentrations adhered to the GB14925-2023 standard (22–24 °C, 12 hour light/dark cycle), with free access to water and food. The study protocol was approved by the Animal Ethics Committee of Hebei Medical University Third Hospital (Z2024-036-2). All procedures followed the “Guide for the Care and Use of Laboratory Animals.” Mice were randomly divided into four groups. Blinding was not performed during the experiment and outcome assessment. Chronic liver injury was induced with 20% CCl₄ (#C805325, Macklin) in olive oil [intraperitoneal (i.p.) injections, 5 mL/kg, twice per week for 12 weeks], followed by combined i.p. injection of 1.0 g/kg D-GalN (#D723130, Macklin) and 100 μg/kg LPS (#00-4976-93, Invivogen) to establish ACLF. Mice in the M8I group additionally received i.p. injections of M8I (#236403-25-1, Cayman; dissolved in PBS containing 10% DMSO) at 2.5 mg/kg for 3 days. Mice in the GW9662 + M8I group additionally received i.p. injections of GW9662 (#HY-16578, MCE; dissolved in olive oil containing 10% DMSO) at 3 mg/kg for 18 days and M8I as described above. Mice in the negative controls (NCs) group received equivalent volumes of olive oil i.p. After mice were anesthetized via i.p. injection of 1.25% Avertin (#LAT-AFD0306, LAT) at 20 μl/g, blood was collected, and fresh liver tissues were harvested post-euthanasia.

### Laboratory assays

Pathological Examination: Liver tissues were subjected to fixation, paraffin embedding, sectioning, deparaffinization, and rehydration sequentially. Hematoxylin-eosin (H&E) staining was performed for histological assessment. Immunohistochemistry (IHC) was conducted with EDTA-mediated antigen retrieval, primary/secondary antibody incubation, 3,3′-diaminobenzidine detection, and semi-quantitative analysis using ImageJ software. Apoptosis was evaluated via a one-step TUNEL assay kit (#KGA1406, KeyGEN). Multiplex immunohistochemistry (mIHC) involved sequential primary/secondary antibody incubation, 4′,6-diamidino-2-phenylindole (DAPI) counterstaining, and multispectral imaging. See Supplementary Table 1 for antibody information.

Nucleic Acid Detection: Total RNA was isolated using TRIzol reagent (#15596026, Thermo Fisher). Quantitative reverse transcription polymerase chain reaction (qRT-PCR) was performed by transcribing RNA into cDNA (#11141ES, Yeasen), followed by PCR amplification and fluorescence signal monitoring (#195199-08-7, Yeasen) to quantitatively analyze RNA expression levels. Primer sequences are listed in Supplementary Table 2. Library construction, sequencing, and subsequent bioinformatics analysis for mRNA-seq were performed by Novogene Bioinformatics Technology Co., Ltd. (Beijing, China).

Protein Detection: Cells were lysed in RIPA buffer (#P0013B, Beyotime) supplemented with 2% protease inhibitors, and total protein concentration was determined by bicinchoninic acid assay (#P0010S, Beyotime). Western blotting (WB) involved protein electrophoresis, transfer to PVDF membranes, and primary/secondary antibody incubation for protein expression detection. Immunofluorescence (IF) included cell fixation, blocking, antibody incubation, and DAPI counterstaining for fluorescent visualization. Co-immunoprecipitation (Co-IP) included cell lysis, incubation of target protein antibody with cell lysate, binding to Protein A + G magnetic beads (#BK0004-02, ACE), elution of immune complexes, and subsequent WB analysis to detect protein interactions. Flow cytometry (FC) involved preparing single-cell suspensions, incubating with CD86 antibody (#11-0862-82, Thermo Fisher) or staining with Annexin V-FITC/PI (#C1062L, Beyotime), followed by detection via a flow cytometer. Scanning electron microscopy (SEM) included cell fixation (#G1102, Servicebio), rinsing, dehydration, critical point drying, conductive coating, and observation under an SEM to assess pyroptotic morphology of M1 macrophages. Enzyme-linked immunosorbent assay (ELISA) was performed to measure serum levels of MMP8, TNF-α, and TGF-β (#EK1M08, EK182HS, EK282HS, EK981; Multisciences). See Supplementary Table 1 for antibody information. All full-length WBs have been compiled into Supplementary Western Blots.

### Statistical analysis

Data were processed with R software (v4.2.0), SPSS (v25.0), GraphPad Prism (v9.1.0), and OmicStudio online tool (https://www.omicstudio.cn/tool). A two-sided *P* < 0.05 was considered statistically significant.

Full and uncropped western blot images are available in the Supplementary Western Blots. See Supplementary Materials for more details.

## Results

### Screening of differentially expressed genes (DEGs) in patients with ACLF

**Fig. 1:**
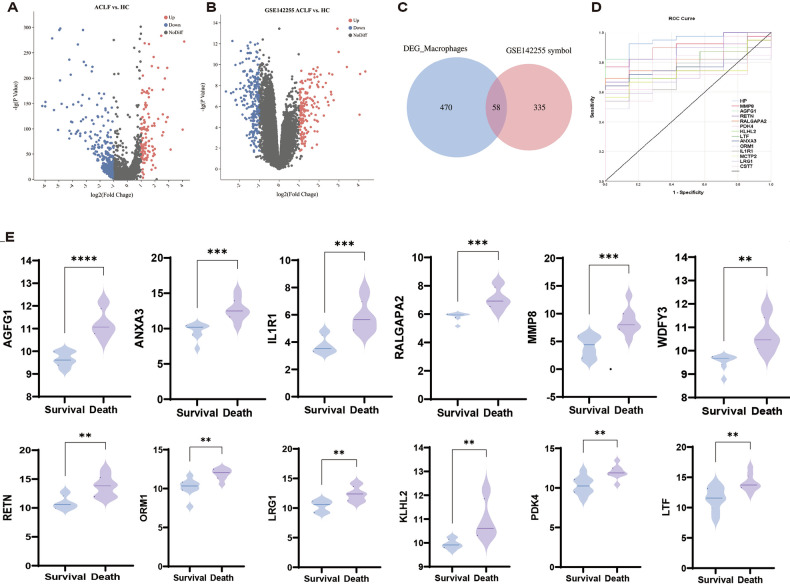
MMP8 is significantly differentially expressed in macrophages of patients with ACLF. **A** Volcano plots of DEGs from the scRNA-seq dataset. **B** Volcano plots of DEGs from the GSE142255 dataset. **C**) Venn diagram showing common DEGs between macrophage-specific DEGs and the GSE142255 datasets. **D** ROC curves of the common DEGs in the GSE142255 dataset. **E** T-test results for common DEGs in the GSE168048 dataset. ACLF acute-on-chronic liver failure, DEGs: differentially expressed genes, ROC: receiver operating characteristic, scRNA-seq: single-cell RNA sequencing.

Liver scRNA-seq data from six patients with ACLF and three HCs, all from the SRA dataset PRJNA913603, were analyzed, including cell clustering and annotation, with a focus on the macrophage population (Supplementary Table 3). Using thresholds of |log₂FC | > 1 and adjusted *P* < 0.05, a total of 528 DEGs were identified in monocytes and macrophages from patients with ACLF, comprising 126 upregulated and 402 downregulated genes (Fig. 1A). Whole-blood transcriptome data from 7 HCs and 17 patients with ACLF in the Gene Expression Omnibus (GEO) dataset GSE142255 [16] were also analyzed, yielding 393 DEGs, including 170 upregulated and 223 downregulated genes (Fig. 1B). A Venn diagram revealed 58 DEGs shared by both datasets (Fig. 1C). Transcriptome data from the GSE142255 dataset were further used to plot receiver operating characteristic (ROC) curves, calculating the area under the curve (AUC) to assess the diagnostic value of these 58 DEGs for ACLF. Fourteen DEGs showed an AUC > 0.70, indicating potential diagnostic value (Fig. 1D). To evaluate prognostic relevance, genome-wide mRNA expression data from PBMCs of eight patients with ACLF (28-day survivors vs. non-survivors) in the GEO dataset GSE168048 [17] were analyzed (Supplementary Table 4). A t-test identified 12 DEGs potentially associated with short-term prognosis (Fig. 1E). Among these, MMP8 exhibited the most pronounced upregulation in both whole blood and PBMCs of patients with ACLF (Supplementary Table 5). Accordingly, MMP8 was selected for subsequent analyses as a candidate gene associated with ACLF diagnosis and prognosis.

### Patients and clinical characteristics

This study enrolled 222 individuals, including 8 HCs, 51 CHB, 40 LC, and 123 HBV-ACLF (19 pre-ACLF, 36 early-stage, 30 intermediate-stage, 38 advanced-stage). The demographic and clinical characteristics of the participants are summarized in Table 1. Laboratory indicators, including neutrophil-to-lymphocyte ratio (NLR), alanine aminotransferase (ALT), total bilirubin (TBil), were significantly increased, whereas albumin (ALB) levels were markedly decreased in the HBV-ACLF group compared with the LC/CHB/HC groups. Serum creatinine (Scr), international normalized ratio (INR), and C-reactive protein (CRP) were significantly increased, and sodium was significantly decreased in the HBV-ACLF group compared with the LC group. Liver transplant-free mortality at 28 and 90 days was 25.96% and 32.69%, respectively, in the HBV-ACLF group.

**Table 1:** Comparison of clinical data between HBV-ACLF, LC, CHB, and HC groups.

Characteristics	HC	CHB	LC	HBV-ACLF			
				Pre	Early	Intermediate	Advanced
Number	8	51	40	19	36	30	38
Year	39 ± 10	41 ± 14	53 ± 12	53 ± 13	50 ± 12	48 ± 10	48 ± 12
M/F(n)	3/5	39/12	31/9	12/7	29/7	25/5	31/7
HBV DNA level (IU/mL)							
≤ 2×10^2^	NA	9(17.65%)	35(87.50%)	16(84.21%)	31(86.11%)	24(80.00%)	27(71.05%)
2×10^2^−2×10^6^	NA	28(54.90%)	5(12.50%)	2(10.53%)	4(11.11%)	4(13.33%)	9(23.68%)
>2×10^6^	NA	14(27.46%)	-	1(5.26%)	1(2.78%)	2(6.67%)	2(5.26%)
MELD	-	-	-	12.27 ± 3.94	15.49 ± 4.66	19.41 ± 5.81	29.14 ± 6.69
MELD-Na	-	-	-	15.34 ± 5.06	18.97 ± 6.19	21.88 ± 5.82	30.83 ± 5.86
COSSH II	-	-	-	5.48 ± 0.91	6.28 ± 0.88	7.11 ± 1.28	8.35 ± 1.18
Lab tests							
WBC (109/L)	5.81 ± 1.53	5.71 ± 1.88	4.65 ± 2.63	4.12 ± 2.42	5.81 ± 3.00	5.44 ± 3.59	8.90 ± 4.84
NLR*	1.91 ± 0.75	1.84 ± 1.01	2.07 ± 1.33	4.80 ± 5.64	5.32 ± 4.05	6.04 ± 4.65	8.89 ± 8.43
ALB* (g/L)	44.40 ± 2.21	41.03 ± 4.43	35.96 ± 7.62	33.53 ± 8.37	30.18 ± 4.59	30.30 ± 5.03	30.33 ± 4.55
ALT* (U/L)	21.38 ± 7.31	197.00 ± 306.85	66.63 ± 92.50	227.27 ± 652.86	39.74 ± 57.48	206.41 ± 258.28	286.92 ± 493.11
AST (U/L)	18.00 ± 2.39	104.69 ± 171.65	63.00 ± 70.14	109.98 ± 172.39	71.42 ± 71.46	204.89 ± 228.35	388.11 ± 1033.92
TBil* (µmol/L)	14.27 ± 2.97	19.16 ± 21.87	43.02 ± 65.94	127.36 ± 82.76	157.20 ± 79.69	231.61 ± 130.94	324.48 ± 139.79
ALP (U/L)	-	107.24 ± 39.18	111.48 ± 72.28	112.08 ± 60.35	188.47 ± 117.22	144.08 ± 59.70	139.76 ± 66.10
GGT (U/L)	-	77.76 ± 71.26	76.03 ± 84.03	84.37 ± 91.99	92.56 ± 161.96	70.13 ± 55.01	75.55 ± 77.20
Scr# (µmol/L)	-	-	69.56 ± 50.35	68.74 ± 22.71	64.21 ± 21.06	70.82 ± 58.95	89.08 ± 65.37
Na+ # (mmol/L)	-	-	138.39 ± 4.59	135.37 ± 4.15	133.79 ± 4.77	134.94 ± 4.77	133.13 ± 6.07
INR#	-	-	1.24 ± 0.18	1.42 ± 0.06	1.72 ± 0.13	2.14 ± 0.29	3.56 ± 1.15
CRP# (mg/L)	-	-	5.23 ± 7.49	14.27 ± 18.08	18.39 ± 22.71	18.62 ± 25.45	16.09 ± 10.91
PCT (ng/ml)	-	-	0.25 ± 0.41	0.38 ± 0.49	0.93 ± 2.40	0.47 ± 0.51	1.87 ± 3.69
Number of deceased patients (transplant-free mortality rate)							
28-day	NA	0	0	0	2(5.56%)	7(23.33%)	18(47.37%)
90-day	NA	0	0	0	3(8.33%)	10(33.33%)	21(55.26%)

*ALB* albumin, *ALP* alkaline phosphatase, *ALT* alanine aminotransferase, *AST* aspartate aminotransferase, *CHB* chronic hepatitis B, *CRP* C-reactive protein, *HBV-ACLF* hepatitis B virus-associated acute-on-chronic liver failure, *HC* healthy control, *INR* international normalized ratio, *LC* liver cirrhosis, *MELD* model for end-stage liver disease, *NLR* neutrophil to lymphocyte ratio, *PCT* procalcitonin, *Scr* serum creatinine, *TBil* total bilirubin, *WBC* white blood cell, Na+, sodium. ^*^ Comparison between HBV-ACLF and LC/CHB/HC group. ^#^ Comparison between HBV-ACLF and LC group.

### MMP8 is associated with ACLF severity and prognosis

**Fig. 2:**
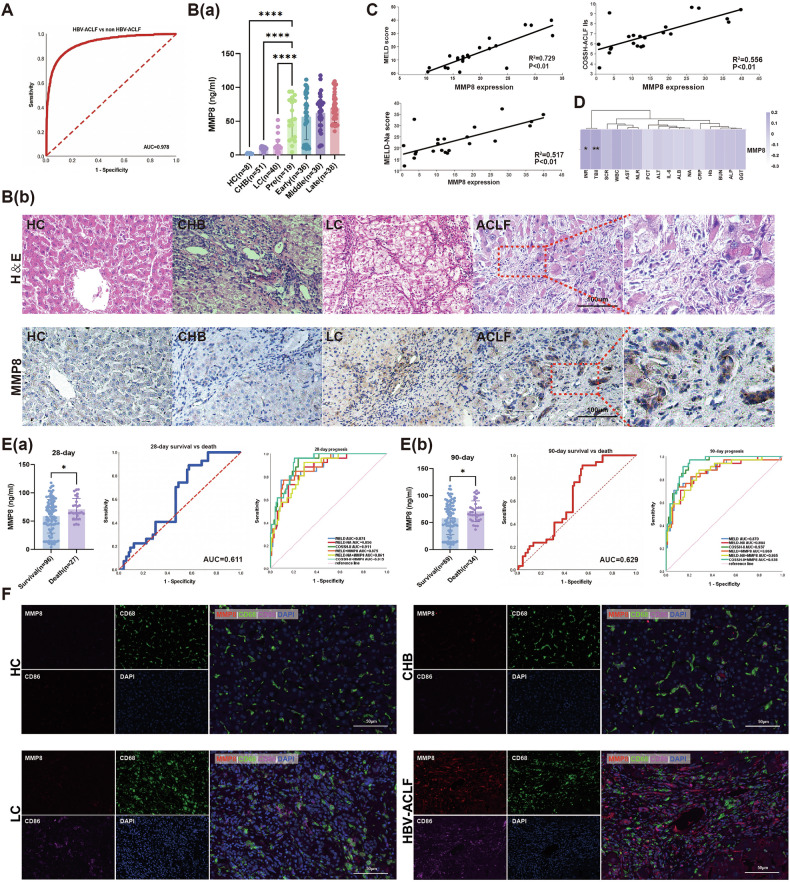
Identification of MMP8 as a potential diagnostic biomarker for HBV-ACLF progression and prognosis. **A** ROC curve for serum MMP8 levels in predicting patients with HBV-ACLF. **B** (a) Serum MMP8 levels in the included population. (b) Representative H&E and MMP8 IHC staining of liver tissues from the included population. **C** Correlation analysis between MMP8 expression in PBMCs and disease severity scores: MELD, MELD-Na, and COSSH-ACLF II. **D** Pearson’s correlation analysis of serum MMP8 levels with key clinical indicators. **E** Serum MMP8 for predicting 28-day (a)/90-day (b) prognosis in patients with HBV-ACLF. **F** Representative mIHC staining of MMP8 (red), CD68 (green), CD86 (purple), and DAPI (blue) in liver tissues from the included population. *P < 0.05, **P < 0.01, ****P < 0.0001. COSSH-ACLF II, Chinese Group on the Study of Severe Hepatitis B–ACLF II, DAPI 4′,6-diamidino-2-phenylindole, HBV-ACLF hepatitis B virus-related acute-on-chronic liver failure, HC healthy control, H&E hematoxylin and eosin, IHC immunohistochemistry, MELD Model for End-Stage Liver Disease, MELD-Na MELD–sodium, MMP8 matrix metalloproteinase 8, PBMCs peripheral blood mononuclear cells, ROC receiver operating characteristic.

MMP8 levels effectively distinguished patients with HBV-ACLF from LC/CHB/HC (AUC = 0.978) (Fig. 2A). Both serum and hepatic MMP8 levels were significantly higher in the HBV-ACLF group than in the LC/CHB/HC groups (Fig. 2B). And notably, MMP8 was significantly increased in the pre-ACLF, and showed an increasing trend with ACLF disease aggravation (Fig. 2Ba). PBMCs were isolated from 22 patients with HBV-ACLF, and qRT-PCR analysis revealed that MMP8 expression was positively correlated with the Model for End-Stage Liver Disease (MELD), MELD–sodium (MELD-Na), and the Chinese Group on the Study of Severe Hepatitis B–ACLF II (COSSH-ACLF II) scores, reflecting disease severity and prognosis (all *P* < 0.01) (Fig. 2C, Supplementary Table 6). Serum MMP8 also positively correlated with liver panel markers, international normalized ratio (INR) and TBil (Fig. 2D). Furthermore, serum MMP8 levels predicted 28/90-day mortality in patients with HBV-ACLF (AUC = 0.611/0.629), with significantly higher levels in non-survivors than in survivors. Notably, the predictive value of the model (MELD, MELD-Na, or COSSH-ACLF II) can be further improved by combining with serum MMP8 (Fig. 2E). mIHC revealed a significantly higher number of CD86⁺/MMP8⁺ macrophages in HBV-ACLF liver tissue than in that of the LC/CHB/HC groups (Fig. 2F).

### Inhibition of MMP8 attenuates M1 macrophage-mediated inflammatory response and prevents hepatocyte apoptosis

**Fig. 3:**
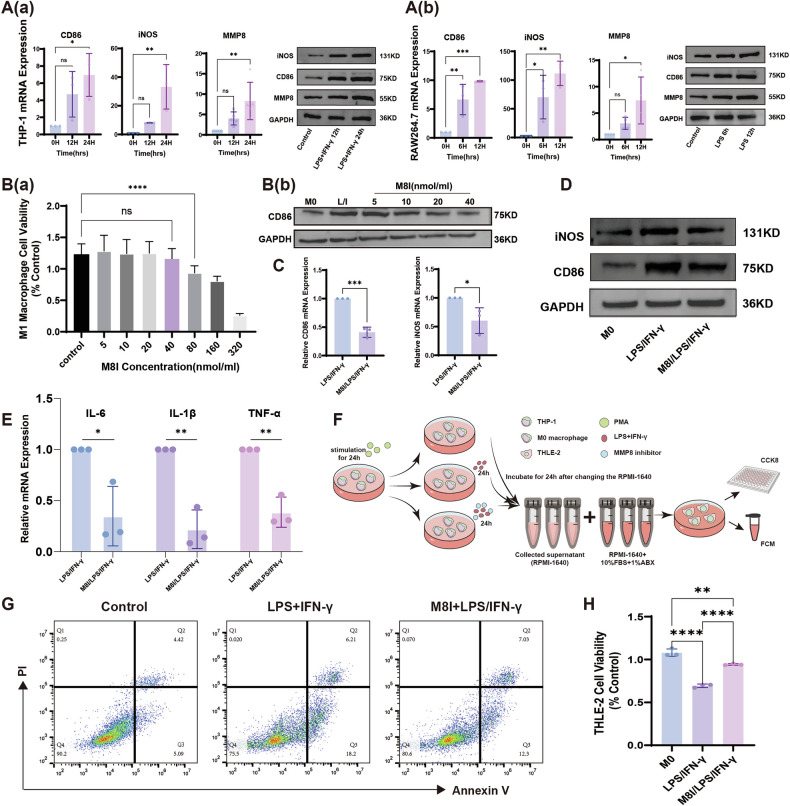
Inhibition of MMP8 reduces macrophage M1 polarization and THLE-2 cell apoptosis. **A** qRT-PCR and WB detection of MMP8, CD86, and iNOS levels during M1 polarization induction in human (a) and mouse (b) macrophages. (**B**) Determination of the optimal MMP8 inhibitory concentration by CCK-8 assay (a) and WB (b). **C**, **D** qRT-PCR and WB detection of M1 macrophage markers CD86 and iNOS expression levels. **E** qRT-PCR detection of inflammatory cytokines IL-6, IL-1β, and TNF-α. **F** Schematic diagram of THLE-2 cell culture in supernatants from different macrophage groups. **G** FC analysis of apoptosis (Q3) in THLE-2 cells treated with macrophage supernatants. **H** CCK-8 assay to detect THLE-2 cell viability after treatment with macrophage supernatants. ns: not significant, *P < 0.05, **P < 0.01, ***P < 0.001, ****P < 0.0001. CCK-8 cell counting kit-8, FC flow cytometry, IL-6 interleukin-6, IL-1β interleukin-1 beta, IFN-γ interferon-gamma, iNOS inducible nitric oxide synthase, LPS lipopolysaccharide, MMP8 matrix metalloproteinase 8, M8I MMP8 inhibitor, qRT-PCR quantitative reverse transcription PCR, THLE-2 immortalized human hepatocyte cell line, TNF-α tumor necrosis factor alpha, WB western blot.

Analysis of publicly available scRNA-seq data confirmed high MMP8 expression in monocytes and macrophages. qRT-PCR and WB assays confirmed that MMP8 expression increased significantly during M1 polarization in both human and mouse macrophages and was accompanied by elevated levels of the M1 macrophage markers CD86 and inducible nitric oxide synthase (iNOS) (Fig. 3A). The CCK-8 assay and WB showed that 40 nmol/mL of M8I was the optimal inhibitory concentration, which did not affect cell viability but significantly inhibited human M1 polarization (Fig. 3B). Therefore, M8I (40 nmol/mL) was chosen for the next experiment. WB and qRT-PCR analyses further confirmed that M8I during M1 polarization significantly reduced both protein and mRNA levels of CD86 and iNOS, as well as mRNA levels of pro-inflammatory cytokines including IL-6, IL-1β, and TNF-α (Fig. 3C–E). Supernatants from M0, LPS/IFN-γ, and M8I/LPS/IFN-γ–treated macrophages were co-cultured with THLE-2 hepatocytes (Fig. 3F). FC and CCK-8 assays showed that THLE-2 cells treated with supernatant from the LPS/IFN-γ group exhibited significantly increased apoptosis and decreased viability relative to controls. In contrast, THLE-2 cells cultured with supernatant from the M8I/LPS/IFN-γ group showed significantly reduced apoptosis and restored viability (Fig. 3G–H).

### MMP8 regulates macrophage polarization by suppressing PPAR-γ

**Fig. 4:**
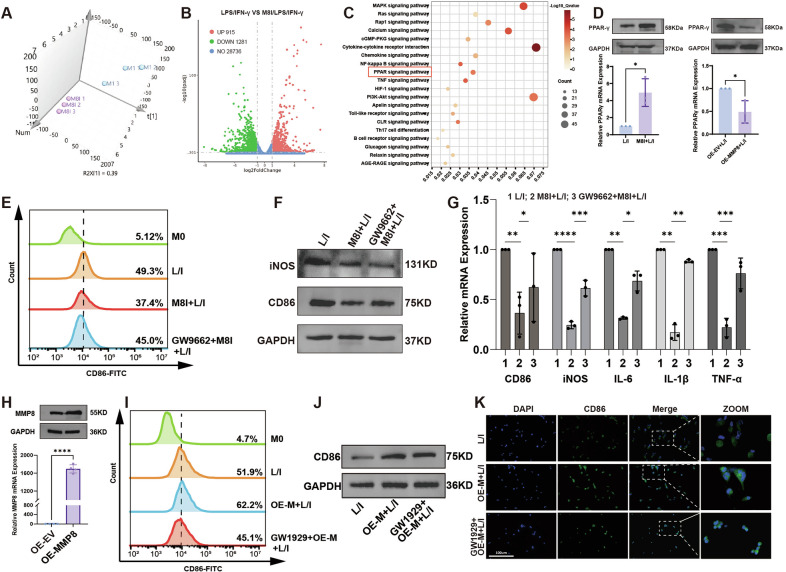
MMP8 regulates M1 macrophage polarization via the PPAR-γ. **A** PCA of transcriptome sequencing for the two cell groups. **B** Volcano plot of DEGs from transcriptome sequencing. **C** KEGG pathway enrichment analysis of DEGs. **D** mRNA and protein levels of PPAR-γ in M1 macrophages after MMP8 inhibition and overexpression. **E** FC detection of CD86 expression in M1 macrophages treated with M8I, with or without GW9662 pretreatment. CD86, iNOS, IL-6, IL-1β, and TNF-α in M1 macrophages treated with M8I, with or without GW9662 pretreatment, determined by WB (**F**) and qRT-PCR (**G**). **H** Verification of MMP8 overexpression by qRT-PCR and WB. CD86 expression in M1 macrophages after MMP8 overexpression, with or without GW1929 pretreatment, measured by FC (**I**), WB (**J**), and IF staining (**K**). LPS and IFN-γ are denoted as L/I. MMP8 overexpression are denoted as OE-M. *P < 0.05, **P < 0.01, ***P < 0.001, ****P < 0.0001. DEGs differentially expressed genes, FC flow cytometry, GW9662 PPAR-γ antagonist, GW1929 PPAR-γ agonist, IF immunofluorescence, IFN-γ interferon gamma, IL-6 interleukin-6, IL-1β interleukin-1 beta, iNOS inducible nitric oxide synthase, KEGG Kyoto Encyclopedia of Genes and Genomes, LPS lipopolysaccharide, MMP8 matrix metalloproteinase 8, M8I MMP8 inhibitor, mRNA messenger RNA, PCA principal component analysis, PPAR-γ peroxisome proliferator-activated receptor gamma, qRT-PCR quantitative reverse transcription PCR, TNF-α tumor necrosis factor-alpha, WB western blot.

Transcriptome sequencing was performed to investigate the mechanism by which MMP8 regulates macrophage phenotypes. Principal component analysis (PCA) showed strong intragroup consistency and clear intergroup separation, confirming the reliability of the differential gene analysis (Fig. 4A). A total of 2,196 DEGs were identified between the M1 and M8I groups, including 915 upregulated and 1,281 downregulated genes, indicating that M8I significantly altered gene expression profiles (Fig. 4B). Kyoto Encyclopedia of Genes and Genomes pathway enrichment analysis revealed that the PPAR signaling pathway was among the top 20 enriched pathways (Fig. 4C). WB and qRT-PCR analyses confirmed that PPAR-γ expression increased following M8I and decreased with MMP8 overexpression (OE-MMP8) (Fig. 4D). Treatment with the PPAR-γ inhibitor GW9662 partially reversed the reductions in M1 macrophage markers (CD86, iNOS) and pro-inflammatory cytokines (IL-6, IL-1β, TNF-α) induced by M8I (Fig. 4E–G). After transfection with an OE-MMP8 plasmid, total RNA and protein were extracted, and successful OE-MMP8 was confirmed by qRT-PCR and WB (Fig. 4H). As expected, treatment with the PPAR-γ agonist GW1929 significantly reduced CD86 expression relative to the OE-MMP8 group (Fig. 4I–K).

### MMP8/PPAR-γ pathway regulates macrophage pyroptosis

**Fig. 5:**
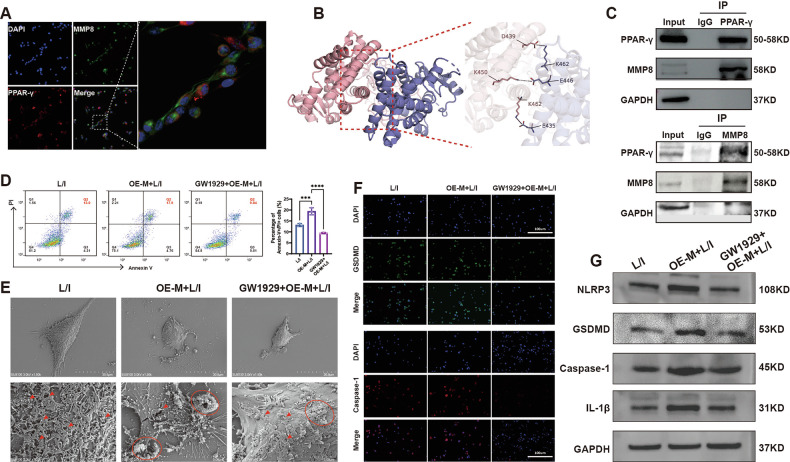
MMP8 overexpression promotes macrophage M1 polarization and pyroptosis. **A** IF detection showing co-localization between MMP8 (green) and PPAR-γ (red). **B** Schematic diagram of protein–protein docking between MMP8 and PPAR-γ. **C** Co-IP analysis of MMP8 and PPAR-γ. **D** FC analysis of cell death (Q2) in M1 macrophages after MMP8 overexpression, with or without GW1929 pretreatment. **E** SEM images: L/I group macrophages showed swelling, formation of vesicles (pyroptosomes), and membrane pores; OE-M + L/I group cells exhibited swelling, collapse, multiple membrane pores, rupture, and content release; GW1929 + OE-M + L/I group displayed reduced swelling, pore formation, and content release. Expression levels of inflammasome component NLRP3 and pyroptosis markers GSDMD, Caspase-1, and IL-1β after MMP8 overexpression, with or without GW1929 pretreatment, detected by IF (**F**) and WB (**G**). LPS and IFN-γ are denoted as L/I. MMP8 overexpression is denoted as OE-M. Co-IP co-immunoprecipitation, FC flow cytometry, GSDMD gasdermin D, IF immunofluorescence, IL-1β, interleukin-1 beta; GW1929, PPAR-γ agonist, MMP8 matrix metalloproteinase 8, NLRP3 NOD-, LRR- and pyrin domain-containing protein 3, PPAR-γ peroxisome proliferator-activated receptor gamma, SEM scanning electron microscopy, WB western blot.

IF images showed co-localization of MMP8 and PPAR-γ in the cytoplasm (Fig. 5A). The 3D structural models of MMP8 (PDB: 1A85) and PPAR-γ (PDB: 8WFE) were retrieved from the Protein Data Bank (PDB; https://www.rcsb.org), and protein–protein docking was performed using the HDOCK server (http://hdock.phys.hust.edu.cn/). The top-ranked binding model exhibited a binding affinity score of –189.54 and a confidence score of 0.6880. PyMOL software (https://pymol.org) was used for complex visualization (Fig. 5B), providing a structural basis for the interaction between MMP8 and PPAR-γ. Co-IP experiments further validated this interaction (Fig. 5C). To assess the functional consequence of this interaction, FC analysis revealed that MMP8 overexpression significantly increased macrophage death, whereas treatment with the PPAR-γ agonist GW1929 significantly reduced cell death (Fig. 5D). SEM observations revealed notable pyroptotic morphological changes in macrophages. Under LPS/IFN-γ stimulation, cells displayed obvious swelling with numerous vesicles (pyroptosomes) and pore formation on the cell membrane. OE-MMP8 further aggravated these morphological alterations, presenting as extensive membrane pore formation, membrane rupture, and intracellular content release. In contrast, treatment with GW1929 effectively alleviated pyroptotic morphological features (Fig. 5E). IF staining and WB analyses of pyroptosis-related signaling pathways demonstrated that OE-MMP8 enhanced LPS/IFN-γ-induced pyroptosis and upregulated the expression of the inflammasome component NLRP3 and pyroptosis markers gasdermin D (GSDMD), caspase-1, and IL-1β. These effects were significantly reversed by GW1929 treatment (Fig. 5F, G).

### PPAR-γ inhibitor reverses the protective effect of M8I in ACLF model mice

**Fig. 6:**
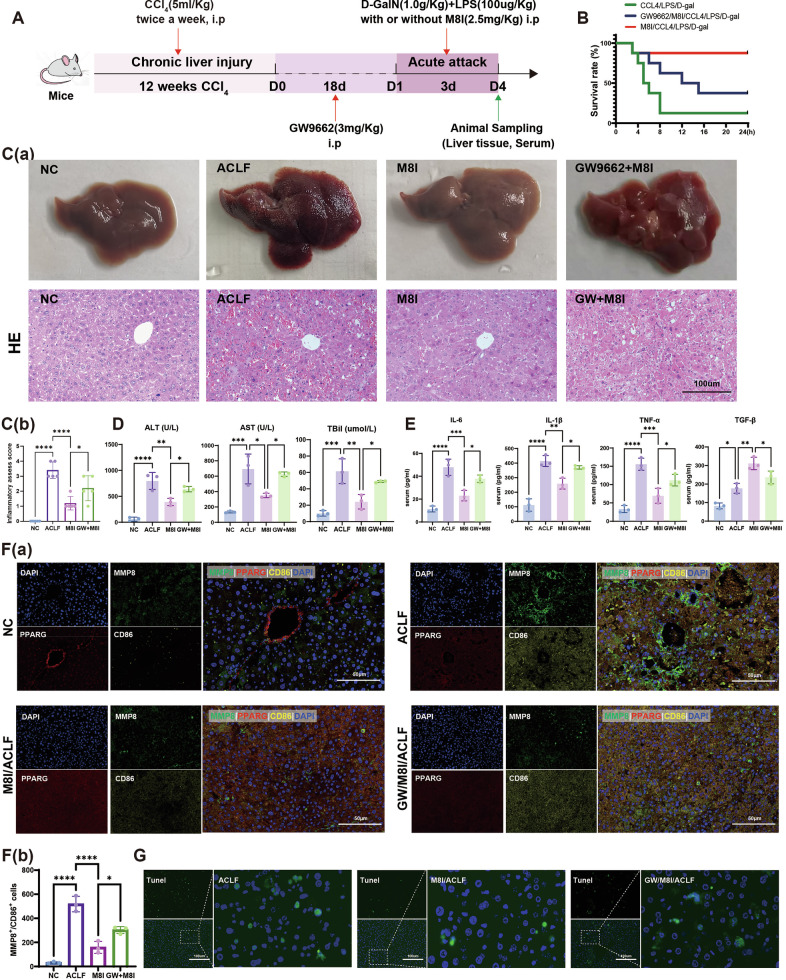
MMP8/PPAR-γ regulates liver injury in ACLF mice. **A** Schematic of the CCl₄/LPS/D-Gal-induced ACLF mouse model and interventions with M8I and GW9662. **B** Survival curves of the three groups over 24 hours. **C** Representative H&E staining of liver tissues and Knodel scores (*n* = 3 mice per group). **D** Liver function indicators in the four groups (*n* = 3 mice per group). **E** ELISA detection of inflammatory cytokines in mouse serum (*n* = 3 mice per group). **F** mIHC staining of MMP8 (green), PPAR-γ (red), CD86 (yellow) and DAPI (blue) in mouse liver tissues from four mice groups. **G** Apoptosis detection (green) in mouse liver tissue. *P < 0.05, **P < 0.01, ***P < 0.001, ****P < 0.0001. ACLF acute-on-chronic liver failure, CCl₄ carbon tetrachloride, DAPI 4′,6-diamidino-2-phenylindole, D-Gal D-galactosamine, ELISA enzyme-linked immunosorbent assay, GW9662 PPAR-γ inhibitor, H&E hematoxylin and eosin, LPS lipopolysaccharide, mIHC multiplex immunohistochemistry, M8I MMP8 inhibitor.

To investigate the role of MMP8 in vivo, mice were treated with 2.5 mg/kg M8I during ACLF modeling (Fig. 6A, Supplementary Table 7). Survival analysis 24 hours after acute LPS/D-GalN challenge showed that mice in the M8I group had significantly higher survival than that of mice in the model group, whereas co-administration of GW9662 (3 mg/kg) partially reversed this protective effect (survival/total: 1/8 vs. 7/8 vs. 5/8, Fig. 6B). Gross examination and H&E staining revealed that mice in the M8I group had reduced liver necrosis, hemorrhage, and inflammatory cell infiltration compared with the ACLF and GW9662 + M8I groups, which was reflected by the Knodel Histological Activity Index score for intralobular portal inflammation (ACLF: 3.40 ± 0.55 vs. M8I:1.20 ± 0.45 vs. GW + M8I:2.20 ± 0.84) (Fig. 6C). Serum biochemical analysis demonstrated that ALT, AST, and TBil levels were significantly lower in the M8I group than in the ACLF group, and GW9662 partially reversed these protective effects (Fig. 6D). ELISA detection showed that serum levels of the pro-inflammatory cytokines IL-6, IL-1β, and TNF-α were significantly lower in the M8I group than in the model group, whereas the level of the anti-inflammatory cytokine TGF-β was significantly higher in the M8I group. GW9662 treatment partially reversed these effects (Fig. 6E).

In vivo, to validate the spatial localization of MMP8 and PPAR-γ and their relationship with M1 polarization in macrophages, we performed mIHC using mouse liver samples from different groups. Consistent with previous studies, mIHC showed double-positive signals for MMP8 and PPAR-γ in the cytoplasm. In addition, the number of MMP8^+^/CD86^+^ cells in the ACLF group was significantly increased compared with the other 3 groups, while the CD86 level in the liver tissue of mice after PPAR-γ inhibition was higher than that in the M8I group (Fig. 6F). TUNEL staining showed significantly fewer apoptotic cells in the M8I group than in the GW9662 group (Fig. 6G).

## Discussion

The pathogenesis of ACLF is complex, with the systemic inflammatory response and immune dysregulation considered key mechanisms [5]. During liver failure, dying hepatocytes release damage-associated molecular patterns (DAMPs), while bacterial infections and gut bacterial translocation generate pathogen-associated molecular patterns (PAMPs). These molecules bind to specific receptors on macrophages, promoting M1 polarization. Polarized macrophages secrete pro-inflammatory mediators, which further induce hepatocyte death and establish a positive hepatotoxic feedback loop between inflammation and cell death [18, 19]. Given the plasticity and multifunctionality of macrophages, identifying key molecules that regulate the differential phenotypes of monocytes and macrophages in ACLF, elucidating their roles, and targeting these molecules to inhibit the production of inflammatory factors and chemokines represent promising therapeutic strategies. In our study, by analyzing public databases and comparing differences in the immune microenvironment between HCs and patients with ACLF, we observed significant differences in monocytes and macrophages in the peripheral blood of patients with ACLF [20] and identified MMP8 as the most significant DEG in monocytes and macrophages, associated with patient prognosis.

MMP8 is a zinc- and calcium-dependent endopeptidase involved in systemic inflammatory responses and macrophage polarization [21], and its levels positively correlate with various systemic inflammatory markers, including C-reactive protein, IL-1α, IL-7, and IL-8 [22]. Previous studies have demonstrated the role of MMP8 in liver fibrosis, cirrhosis, and hepatocellular carcinoma, as well as its association with the severity and mortality of systemic inflammatory diseases such as sepsis [23]. However, its role in ACLF onset and prognosis remains unclear. In this prospective observational study, we validated that MMP8 was significantly elevated in the liver tissue, serum, PBMCs, and CD86^+^ macrophages of patients with HBV-ACLF, particularly in 90-day non-survivors, with high sensitivity and specificity. MMP8 levels increased progressively with ACLF stage, indicating its potential as an early-warning biomarker. Furthermore, combining serum MMP8 levels with MELD, MELD-Na, or COSSH-ACLF II scores effectively enhanced the predictive performance of these models. Strong systemic inflammation, primarily triggered by infection and extensive hepatocyte injury, is a key driver of ACLF development [19, 24]. In this study, during M1 polarization induction in THP-1 and RAW264.7 cells, increased expression of the M1 markers CD86 and iNOS was accompanied by progressively elevated MMP8 levels and significantly increased production of the inflammatory cytokines (IL-6, IL-1β, and TNF-α). Conversely, inhibiting MMP8 significantly reduced these cytokines in macrophages. Co-culturing THLE-2 hepatocytes with supernatant from MMP8-inhibited macrophages reduced hepatocyte apoptosis, indicating that MMP8 contributes to both inflammatory activation and hepatocyte injury. Transcriptome sequencing further revealed that MMP8 inhibition modulates macrophage M1 polarization and inflammatory cytokine release through the PPAR pathway, highlighting a potential mechanistic target for developing novel therapeutic strategies.

PPARs are highly expressed in the liver and participate in multiple physiological and pathological processes, including oxidative stress and inflammation. They regulate macrophage phenotypic switching and pyroptosis, both of which are closely associated with the progression of liver diseases [25, 26]. In a neuroinflammatory disease model, M8I was shown to increase LPS-induced NF-κB, MAP kinase, and Akt activity while simultaneously enhancing the activity of the anti-inflammatory mediator PPAR-γ, leading to a significant reduction in the release of pro-inflammatory factors and reactive oxygen species production [27]. Additionally, a previous multicenter prospective clinical cohort study demonstrated that the expression of the pyroptosis-specific marker GSDMD was significantly higher in the liver tissue of patients with ACLF than in those with acutely decompensated cirrhosis. This upregulation was particularly evident in patients with concurrent infections, in whom GSDMD expression correlated strongly with systemic inflammatory response syndrome. GSDMD staining intensity was also significantly higher in non-survivors than in survivors, and its expression level positively correlated with the chronic liver failure–consortium organ failure score, consistent with its prognostic relevance in plasma [28]. NLRP3 is the most extensively studied inflammasome and has been widely implicated in the pathology of various inflammatory diseases, where it drives immune-cell activation and amplification. Previous studies have shown a negative correlation between PPAR-γ and NLRP3 inflammasome activation, and PPAR-γ is required for suppressing DAMP-induced NLRP3 activation in macrophages [29]. Integrating these findings, our study demonstrates that MMP8 interacts with PPAR-γ, promotes macrophage M1 polarization and pro-inflammatory cytokine release, and induces M1 macrophage pyroptosis, thereby contributing to the vicious cycle of inflammation and cell death in ACLF pathogenesis. To model ACLF, we used CCl₄ to induce chronic liver injury and LPS/D-GalN to simulate an acute PAMP- and DAMP-mediated insult. M8I alleviated CCl₄/LPS/D-GalN-induced liver failure, improved liver function, reduced systemic inflammation, decreased macrophage M1 polarization, and reduced hepatocyte apoptosis. Notably, when PPAR-γ activity was inhibited, the protective effect of M8I on systemic inflammation was partially reversed, further confirming that the MMP8–PPAR-γ axis mediates ACLF progression.

Given the complex, multifactorial, and multilevel pathophysiological processes of ACLF, the development of novel targeted drugs with anti-inflammatory and liver regeneration–promoting effects remains a research priority. Current evidence shows that phillygenin, an active component of Forsythia suspensa, activates the PPAR-γ signaling pathway by downregulating MMP8, thereby inhibiting inflammation and apoptosis in lung epithelial cells [30]. PPAR agonists, including those targeting PPAR-α, PPAR-γ, and PPAR-δ, are increasingly used in mechanistic and clinical studies to regulate metabolism, inflammation, or fibrosis. Wu et al. [31] reported that the antioxidant quercetin directly binds to PPAR-γ, significantly inhibits the NF-κB pathway, promotes mitophagy, and suppresses apoptosis and inflammatory responses mediated by mitochondrial dysfunction, thereby improving liver failure. Additionally, geraniol ameliorates the progression of acute liver failure by inhibiting PPAR-γ methylation and suppressing NLRP3 inflammasome-mediated inflammation in M1 macrophages [32]. Wei et al. [33] found that NLRP3 impairs liver regeneration by inhibiting MerTK-mediated efferocytosis and reparative phenotypic switching in macrophages. Overall, these findings provide a new perspective on the therapeutic mechanism of targeting MMP8 in ACLF and highlight the potential value of the MMP8/PPAR-γ axis as a promising treatment strategy, warranting further exploration of its clinical applicability.

This study has some limitations. First, the foundational evidence supporting the interaction between MMP8 and PPAR-γ is not fully comprehensive. Future work should include assays such as GST pull-down, truncated mutant analyses, and functional studies following the interaction. Second, evidence for the MMP8/PPAR-γ pathway in regulating macrophage polarization and modulating the immune–inflammatory response in vivo remains insufficient. Future studies using macrophage-specific PPAR-γ knockout mouse models are required to directly elucidate these mechanisms.

In conclusion, this study systematically demonstrated that MMP8 is highly expressed in patients with HBV-ACLF and provides accurate diagnostic and prognostic value for HBV-ACLF. MMP8 exacerbates ACLF progression by inhibiting PPAR-γ, leading to macrophage-mediated systemic inflammatory responses and hepatocyte apoptosis. As a key regulator of macrophage-driven inflammation in ACLF, MMP8 represents a promising target for early therapeutic intervention.

## Supplementary information

Supplementary Materials

Supplementary Western blots

## Data Availability

All data are included in the Supplementary Information or available from the authors upon reasonable requests (Caiyan Zhao, zhaocy@hebmu.edu.cn).
