# Supplementary material for: MMP8/PPAR-γ regulation of macrophage-mediated inflammatory response in the pathogenesis of acute-on-chronic liver failure
Source: Cell Death Dis. 2026 Apr 27;17(1):556. doi: 10.1038/s41419-026-08793-z (PMC13249895; doi:10.1038/s41419-026-08793-z)
Supplement: Supplementary file 1 — Supplementary Materials [file 41419_2026_8793_MOESM1_ESM.docx]

**Supplementary material**

**Contents**

**Methods**

Differential gene screening and processing

Clinical study and specimen processing

Cell culture and in vitro intervention

ACLF mouse model construction and grouping

Laboratory assays

Statistical analysis

**Tables**

Table S1 List of antibodies used in experiments

Table S2 Primer sequences for RT-PCR

Table S3 scRNA datasets

Table S4 DEGs expression in GSE168048 dataset

Table S5 logFC values of 12 DEGs in ACLF/NC group or Death/Survival group

Table S6 Scores and MMP8 expression level in PBMC of 22 HBV-ACLF patients

Table S7 Concentration of M8I determined by preliminary experiments

**Methods**

**Differential gene screening and processing**

ACLF-related datasets were retrieved from the GEO database using the keyword "acute-on-chronic liver failure.” Datasets that included both normal controls and ACLF samples were selected. After quality control to ensure suitability for differential analysis, microarray data were preprocessed using GEO2R, and probe IDs were converted to their corresponding gene names. The processed datasets were then normalized and subjected to differential expression analysis in R software (v4.2.0). DEGs were identified using the thresholds |log₂(fold change, FC)| ≥ 1 and adjusted P < 0.05. Volcano plots were generated to visualize the results. The pROC and ggplot2 packages were used to evaluate the diagnostic performance of DEGs for ACLF by plotting ROC curves and calculating the AUC. Genes with AUC ≥ 0.70 (P < 0.05) were considered potential biomarkers. GEO datasets containing patients’ prognosis information were retrieved for external validation. Differences in DEG expression between survival and non-survivors in the validation set were assessed using two-sided t-tests, with P < 0.01 considered statistically significant.

scRNA-seq data from six patients with ACLF and three HCs were downloaded from the SRA project PRJNA913603. CellRanger (v7.1.0) was used for sample demultiplexing, barcode processing, and generation of single-cell gene–Unique Molecular Identifier (UMI) count matrices, aligning reads to the GRCh38 reference genome. Seurat (v5.0.1) was used for data quality control and filtering, retaining cells with at least 200 detected genes (nFeature_RNA ≥ 200), total UMI counts (nCount_RNA) below the 95th percentile, and mitochondrial transcript content ≤ 15%. The integrated dataset was then used for clustering, cell-type annotation, and differential expression analyses. For macrophages, genes with P < 0.05 and |log₂FC| > 1 were defined as DEGs.

**Clinical study and specimen processing**

HCs from the Physical Examination Center and hospitalized patients with CHB, LC and HBV-ACLF were consecutively enrolled at Hebei Medical University Third Hospital between October 2023 and April 2025. LC refers to hepatitis B virus-infected cirrhotic patients. Diagnoses of ACLF, LC and CHB followed the guidelines of the Asian Pacific Association for the Study of the Liver (PMID: 31172417), European Association for the Study of the Liver (PMID: 29653741) and American Association for the Study of Liver Diseases (PMID: 19714720). The estimated sample size ratio between the non-ACLF and ACLF groups was set at 1:1. With a two-sided type I error margin of 7% and a target confidence level of 95%, a minimum of 196 patients were required for the study. ACLF was also divided into pre, early, intermediate, and advanced stages according to jaundice and coagulation in ACLF patients (PMID: 41334648). Pre-ACLF: 1) severe gastrointestinal symptoms; 2) markedly elevated ALT and/or AST levels with progressive hyperbilirubinemia (TBil 5–12 mg/dL [85.5–205.2 µmol/L]); and 3) coagulopathy, progressive decrease in PTA but > 40% and INR < 1.5. Early stage: 1) severe gastrointestinal symptoms; 2) elevated ALT and/or AST levels accompanied by progressive hyperbilirubinemia (TBil ≥12 mg/dL [205.2 µmol/L] or daily increment ≥1 mg/dL [17.1 µmol/L]), and 3) incipient coagulopathy (30% <PTA ≤40% or 1.5 ≤INR <1.9). Intermediate stage: Worsening hyperbilirubinemia, aggravated coagulopathy (20% <PTA ≤30% or 1.9 ≤INR <2.5), and progressive clinical deterioration from early-stage features. Advanced stage: PTA ≤ 20% (INR ≥ 2.5); or further aggravation of the disease, severe bleeding tendency, grade 3 ~ 4 hepatic encephalopathy, renal failure and other serious complications. Exclusion criteria were: 1) ACLF caused by etiologies other than HBV infection, 2) presence of liver or extrahepatic solid organ malignancies, and 3) incomplete clinical data. The study protocol was approved by the Medical Ethics Committee of Hebei Medical University Third Hospital (No: K2025-156-1). All the procedures were conducted in accordance with the principles of the Declaration of Helsinki, and written informed consent was obtained from all participants before enrollment.

Demographic data (age and sex) and laboratory test results, including WBC, liver and kidney function parameters, coagulation profile, and inflammatory markers, were collected from all participants. Patients received standard clinical treatment and were followed up for 28/90 days to determine clinical outcomes. The NLR, MELD score, and COSSH-ACLF II score were calculated based on the clinical and laboratory indicators.

Specimen collection and processing: Peripheral venous blood samples were collected from all participants. For serum, blood was drawn into tubes containing an inert separation gel and coagulant, then centrifuged at 4000 rpm for 5 minutes at room temperature within 8 hours. The supernatant was collected and stored at −80 °C until analysis. For PBMC isolation, blood was collected into EDTA-anticoagulated tubes, diluted 1:1 with phosphate-buffered saline (PBS), carefully layered over an equal volume of lymphocyte separation medium (#LTS10771, TBD), and centrifuged at 2000 rpm for 20 minutes. PBMCs were then collected from the interface. Liver tissues were obtained from patients with HBV-ACLF undergoing liver transplantation, those with CHB undergoing liver biopsy, and donor liver trimming as normal liver tissue controls.

**Cell culture and in vitro intervention**

Cell culture: The human monocyte cell line THP-1 (#CL-0233, Procell) was cultured in RPMI-1640 medium (#12633020, Thermo Fisher Scientific) supplemented with 20% fetal bovine serum (FBS, #10099141C, Thermo Fisher Scientific) and 1% penicillin–streptomycin (#15140122, Thermo Fisher Scientific). THP-1 cells were seeded at a density of 1 × 10⁶ cells per well in 6-well plates, and then differentiated into macrophages with 100 ng/mL phorbol 12-myristate 13-acetate (PMA, #HY-18739; MCE). After 24 hours, the medium was replaced, and cells were stimulated with 100 ng/mL LPS (#HY-D1056, MCE) and 20 ng/mL IFN-γ (#DC054, Novoprotein) for 0, 12, or 24 hours before RNA and protein extraction. The mouse macrophage cell line RAW264.7 (Cell Bank, Chinese Academy of Sciences) was cultured in DMEM (#11965084, Thermo Fisher Scientific) supplemented with 10% FBS and 1% antibiotics. Cells were stimulated with 200 ng/mL LPS for 0, 6, or 12 hours prior to harvesting for RNA and protein extraction. The immortalized human hepatocyte cell line THLE-2 (#iCell-h388, iCell) was cultured in the manufacturer-provided medium (#iCell-h388-001b). Human embryonic kidney 293T cells (#iCell-h477, iCell) were cultured in DMEM supplemented with 10% FBS and 1% antibiotics. All cell lines were maintained at 37 °C in a humidified 5% CO₂ incubator.

Cell viability assay: Cell proliferation was assessed using CCK-8 (#RC3028, ReportBio). Human M0 macrophages were seeded into 96-well plates at a density of 1 × 10⁴ cells per well and then treated with 100 ng/mL LPS, 20 ng/mL IFN-γ, and varying concentrations of M8I (0, 5, 10, 20, 40, 80, 160, and 320 nmol/mL). After 24 hours, 10 μL of CCK-8 solution was added to each well, followed by incubation for 2 hours. Absorbance was measured at 450 nm using a microplate reader (#keebio-MR100, JiaPeng Technology Co., Ltd.), and cell viability was calculated accordingly.

Cell transfection and treatment: MMP8 and PPAR-γ overexpression plasmids (pCDNA3.1^+^) were synthesized by Beijing Bomaide Biotechnology Co., Ltd (Beijing, China). THP-1-derived M0 macrophages and 293T cells were seeded in 6-well plates at a density of 1 × 10⁴ cells/mL. Overexpression plasmids were transfected using Lipofectamine 2000 (#11668019, Thermo Fisher Scientific). Gene expression was evaluated by qRT-PCR and WB after 24 and 48 hours of culture. For PPAR-γ modulation, M0 macrophages were pretreated with 10 μM GW1929 (#HY-15655, MCE) to activate or 10 μM GW9662 (#HY-16578, MCE) to inhibit PPAR-γ expression for 12 or 24 hours, respectively, before subsequent stimulation.

**ACLF mouse model construction and grouping**

Housing and grouping: Forty-five 3–4-week-old male BALB/c mice were purchased from Beijing Huafukang Bioscience Co., Ltd. (Certificate No: SCXK (Jing) 2024-0003) and housed in a standard-specific pathogen-free environment. Temperature, humidity, light/dark cycle, and noise/ammonia concentrations adhered to the GB14925-2023 standard (22–24 °C, 12 hour light/dark cycle), with free access to water and food. The study protocol was approved by the Animal Ethics Committee of Hebei Medical University Third Hospital (Z2024-036-2). All procedures followed the "Guide for the Care and Use of Laboratory Animals.” Mice were randomly divided into four groups.

1) ACLF model group: (a) Chronic injury phase: Mice received intraperitoneal (i.p.) injections of 20% CCl₄ (#C805325, Macklin) in olive oil, 5 mL/kg, twice per week for 12 weeks, resulting in microscopically visible fibrous tissue hyperplasia, pseudolobule formation, and disordered hepatocyte plates in regenerative nodules. (b) Acute attack phase: Six hours after the last CCl₄ injection, mice received a combined i.p. injection of 1.0 g/kg D-GalN (#D723130, Macklin) and 100 μg/kg LPS (#00-4976-93, Invivogen).

2) MMP8 Inhibitor (M8I) group: Chronic injury phase was identical to the model group. Three days before the acute attack, mice received daily i.p. injections of M8I (#236403-25-1, Cayman; dissolved in PBS containing 10% DMSO) at 2.5 mg/kg. Six hours after the last M8I injection, an acute attack was induced with the same doses of D-GalN and LPS.

3) PPAR-γ Inhibitor (GW9662) + M8I group: Chronic injury phase was identical to the model group. Eighteen days before the acute attack, mice received daily i.p. injections of GW9662 (#HY-16578, MCE; dissolved in olive oil containing 10% DMSO) at 3 mg/kg. Three days before the acute attack, M8I was administered as described above. Six hours after the last injection, the acute attack was induced as in the model group.

4) HC group: Received equivalent volumes of olive oil i.p. twice weekly for 12 weeks.

Specimen collection: Eight hours after the final injection in all groups, mice were anesthetized via i.p. injection of 1.25% Avertin (2,2,2-Tribromoethanol, #LAT-AFD0306, LAT) at 20 μl/g. Blood was collected via cardiac puncture, left at room temperature for 2 hours, and then centrifuged at 3500 rpm for 10 minutes at 4 °C. The resulting supernatant (serum) was collected and stored at –80 °C for subsequent biochemical and cytokine analyses. Fresh liver tissues were harvested post-euthanasia and fixed in 10% formalin for histological evaluation.

**Laboratory assays**

Pathological examination

Liver tissues were fixed, paraffin-embedded, and sectioned. Sections were deparaffinized and rehydrated through a graded alcohol series for subsequent staining procedures.

H&E staining: Sections were stained with hematoxylin to visualize nuclei, differentiated with hydrochloric acid alcohol, and counterstained with eosin for the cytoplasm. The sections were then dehydrated through graded alcohols, cleared, and mounted for microscopy.

IHC staining: After antigen retrieval using EDTA (#ZLI-9068, ZSGB-BIO) and blocking of endogenous peroxidase activity (#PV-9000, ZSGB-BIO), sections were incubated with primary antibodies, followed by incubation with a secondary antibody (Supplementary Table 1). Detection was performed using 3,3′-diaminobenzidine, and sections were counterstained with hematoxylin. Dehydration, clearing, and mounting were performed, and images were captured using a ZEISS Axioscope 5 microscope. Brown-yellow staining indicated positive expression. Semi-quantitative analysis was conducted using ImageJ (v1.48) software.

Cell apoptosis detection: Apoptosis in tissue samples was assessed using a one-step TUNEL assay kit (#KGA1406, KeyGEN) according to the manufacturer's instructions. Images were captured using an IX51 fluorescent inverted microscope (Olympus, Tokyo, Japan).

mIHC Staining: After EDTA antigen retrieval, tissue sections were washed with Tris-buffered saline containing Tween 20 and blocked with an immunostaining blocking buffer. Sections were then sequentially incubated with the primary antibody (Supplementary Table 1), a universal secondary antibody, and the corresponding fluorescent dye. This process was repeated for all the antibodies. Following 4′,6-diamidino-2-phenylindole (DAPI) staining, the sections were mounted. Images were acquired using an Akoya PhenoImager-Fusion multispectral scanner and visualized with Phenochart software.

Nucleic acid detection

qRT-PCR: Total RNA was isolated from cells using TRIzol reagent (#15596026, Thermo Fisher). cDNA synthesis was performed using 5× gDNA digester buffer, 4× Hifair® SuperMix, and RNase-free H₂O (#11141ES, Yeasen) in a metal water bath. Using the synthesized cDNA as a template, gene-specific primers and iTaq™ universal SYBR® Green supermix (#195199-08-7, Yeasen) were used for PCR amplification on a 7500 Real-Time PCR System (Applied Biosystems). Relative mRNA expression levels were calculated using the 2^(-ΔΔCT)^ method. Primer sequences are listed in Supplementary Table 2.

mRNA-seq: RNA quantity and purity were assessed using an RNA 6000 Nano LabChip kit (#5067-1511, Agilent Technologies) and a Bioanalyzer 2100. Library construction and sequencing were performed by Beijing Novogene Bioinformatics Technology Co., Ltd. (Beijing, China), followed by bioinformatics analysis on the obtained data.

Protein detection

WB: Cell samples were lysed using RIPA lysis buffer (#P0013B, Beyotime) supplemented with 2% protease inhibitors. Total protein concentration was determined with a bicinchoninic acid (BCA) assay (#P0010S, Beyotime). Proteins were mixed with 5× sodium dodecyl sulfate–polyacrylamide gel electrophoresis (SDS-PAGE) loading buffer (#P0015L, Beyotime), separated by electrophoresis, and transferred onto polyvinylidene fluoride (PVDF) membranes. Membranes were blocked using 5% powdered milk (#LP0033B, Solarbio) in Tris-buffered saline comprising Tween 20 (#T1085, Solarbio) at room temperature for 1 h. Subsequently, the membranes were sequentially incubated with primary and secondary antibodies (Supplementary Table 1). Protein signals were visualized using an enhanced chemiluminescence detection system (#SW134, Seven), and images were captured using a chemiluminescence imaging system (#JP-K600, JiaPeng Technology Co., Ltd.).

IF: Cells were fixed with 4% paraformaldehyde and blocked with 5% bovine serum albumin (BSA; #SW3015, Solarbio). They were then incubated with primary antibodies for 2 hours or overnight, washed with PBS, and incubated with fluorescent secondary antibodies in the dark for 1 hour (Supplementary Table 1). An anti-fade mounting medium containing DAPI (#P0131, Beyotime) was used, and images were captured using an Olympus IX51 fluorescence microscope.

Co-IP: 293T cells overexpressing MMP8 or PPAR-γ were lysed in immunoprecipitation (IP) lysis buffer at 4 °C for 10 minutes, followed by centrifugation at 12,000×*g* for 10 minutes. Protein concentrations in the supernatant were determined using a BCA assay. A portion of the supernatant was mixed with SDS loading buffer and heated at 100 °C for 15 minutes to serve as the input group. A total of 1000 μg of cell protein was incubated with 5 μg of either MMP8 or PPAR-γ antibody, or IgG control antibody, overnight at 4 °C with gentle rotation. Subsequently, 0.2 mg of Protein A+G magnetic beads (#BK0004-02, ACE) were added and incubated at 4 °C for 6 hours with rotation. Co-immunoprecipitated proteins were eluted using acidic elution buffer at room temperature for 10 minutes with rotation. SDS loading buffer was then added, and the samples were boiled for 15 minutes and centrifuged at 12,000 × g for 10 minutes, and the supernatant was collected for downstream WB analysis.

FC: For macrophage marker detection, cells from each group were prepared as single-cell suspensions (~10⁶ cells), incubated with diluted CD86 antibody (#11-0862-82, Thermo Fisher) with gentle mixing, and kept on ice in the dark for 20 minutes. For cell death and apoptosis analysis, cell suspensions were sequentially mixed with 195 μL Annexin V-FITC binding buffer, 5 μL Annexin V-FITC, and 10 μL propidium iodide staining solution (#C1062L, Beyotime), followed by incubation at room temperature in the dark for 20 minutes. Samples were analyzed within 1 hour using a CytoFLEX LX flow cytometer (Beckman Coulter).

SEM: Cells grown on coverslips were rinsed with PBS and fixed with electron microscopy fixative (#G1102, Servicebio) at room temperature for 2 hours. After rinsing with 0.1 M phosphate buffer (PB, pH 7.4), samples were further fixed with 1% osmium tetroxide at room temperature in the dark for 2 hours. The cells were then dried using a critical point dryer, coated with a conductive material using an ion sputter coater, and observed under a Hitachi SU8100 SEM to assess the pyroptotic morphology of M1 macrophages across different treatment groups.

ELISA: MMP8, TNF-α, and TGF-β levels in human or mouse serum were measured using the respective ELISA kits (#EK1M08, EK182HS, EK282HS, EK981; Multisciences). Absorbance was measured at 570 nm using a microplate reader (#keebio-MR100, JiaPeng Technology Co., Ltd.).

**Statistical analysis**

Continuous variables were expressed as mean ± standard deviation or median (25th, 75th percentile), while categorical variables were expressed as number (percentage). Data were analyzed using R software (v4.2.0), SPSS (v25.0) and GraphPad Prism (v9.1.0). Comparisons between two groups were performed using Student's t-test or the Mann–Whitney U test, as appropriate. Multigroup comparisons of continuous variables were conducted using the Kruskal–Wallis test, and categorical variables were compared using the χ² test and Fisher's exact test. ROC curves were plotted, and the AUC was calculated, with AUC values of 0.6–0.9 indicating diagnostic accuracy and values > 0.9 indicating high diagnostic ability. Spearman’s correlation analysis was performed using the OmicStudio online tool (https://www.omicstudio.cn/tool). A two-sided p < 0.05 was considered statistically significant.

**Tables**

Table S1 List of antibodies used in experiments

| Antigen | Cat. | Source | Applications |
| --- | --- | --- | --- |
| Primary antibodies | | | |
| MMP8 | YT2800 | Inmunoway | WB, IF, IHC, co-IP |
| CD68 | ER1901-32 | HUABIO | IF |
| CD86 | ET1606-50 | HUABIO | WB, IF |
|  | 11-0862-82 | Thermo Fisher | FC |
| iNOS | ER1706-89 | HUABIO | WB |
| CD80 | YA3386 | MCE | IHC |
| F4/80 | HA721520 | HUABIO | IHC |
| PPARγ | 16643-1-AP | Proteintech | WB, co-IP |
|  | 66936-1-1g | Proteintech | IF |
| NLRP3 | 30109-1-AP | Proteintech | WB |
| Caspase-1 | ET1608-69 | HUABIO | WB, IF |
| GSDMD | ER1901-37 | HUABIO | WB, IF |
| IL-1β | ET1701-39 | HUABIO | WB |
| GAPDH | ET1601-4 | HUABIO | WB |
| Mouse IgG | AC011 | ABclonal | co-IP |
| Rabbit IgG | AC005 | ABclonal | co-IP |
| Secondary antibodies |  |  |  |
| anti-Mouse IgG HRP | HA1006 | HUABIO | WB |
| anti-Rabbit IgG HRP | HA1001 | HUABIO | WB |
| anti-Rabbit IgG FITC | HA1004 | HUABIO | IF |
| anti-mouse IgG cy3 | HA1109 | HUABIO | IF |
| anti-Rabbit IgG (Light Chain)-HRP | SA00001-7L | Proteintech | co-IP |
| Anti-rabbit IgG for IP (HRP) | RA1008-01 | Vazyme | co-IP |

*WB, Western Blotting; IP, Immunoprecipitation; IF, Immunofluorescence; IHC, Immunohistochemistry; FC, Flow Cytometry.

Table S2 Primer sequences for RT-PCR

| Host | Gene | Forward primer（5’-3’） | Reverse primer（5’-3’） |
| --- | --- | --- | --- |
| HOMO | MMP8 | CAACCTACTGGACCAAGCACAC | TGTAGCTGAGGATGCCTTCTCC |
|  | CD86 | CTGCTCATCTATACACGGTTACC | GGAAACGTCGTACAGTTCTGTG |
|  | β-actin | CATGTACGTTGCTATCCAGGC | CTCCTTAATGTCACGCACGAT |
| Mouse | MMP8 | GATGCTACTACCACACTCCGTG | TAAGCAGCCTGAAGACCGTTGG |
|  | CD86 | TGTTTCCGTGGAGACGCAAG | TTGAGCCTTTGTAAATGGGCA |
|  | iNOS | ACATCGACCCGTCCACAGTAT | CAGAGGGGTAGGCTTGTCTC |
|  | IL-6 | TAGTCCTTCCTACCCCAATTTCC | TTGGTCCTAGCCACTCCTTC |
|  | IL-1α | TGATGAAGCTCGTCAGGCAG | GCAACTCCTTCAGCAACACG |
|  | IL-1β | GCTTCAGGCAGGCAGTATCA | AGTCACAGAGGATGGGCTCT |
|  | TGF-β | CGTAAGACATTCGGGAAGCA | ACTGCCGTACAACTCCAGTG |
|  | β-actin | GGCTGTATTCCCCTCCATCG | CCAGTTGGTAACAATGCCATGT |

Table S3 scRNA datasets

| Database ID | Name | Source | Before QC | QC filtering low qulaity cells | After QC | All |
| --- | --- | --- | --- | --- | --- | --- |
|  |  |  | # cells |  | # of cells |  |
| SRR22804964 | ACLF1 | Liver | 4007 | nFeature_RNA>=200 nCount_RNA<=95%  quantile mitochondrial gene % <=15% | 3483 | 19583 |
| SRR22804960 | ACLF2 | Blood | 2260 |  | 1862 |  |
| SRR22804963 | ACLF3 | Liver | 2739 |  | 2472 |  |
| SRR22804959 | ACLF4 | Liver | 3984 |  | 3474 |  |
| SRR22804958 | ACLF5 | Liver | 6560 |  | 6115 |  |
| SRR22804957 | ACLF6 | Liver | 2514 |  | 2177 |  |
| SRR22804953 | HC1 | Liver | 4252 |  | 2777 | 9482 |
| SRR22804952 | HC2 | Liver | 1420 |  | 1302 |  |
| SRR27193253 | HC3 | Liver | 5688 |  | 5403 |  |

Table S4 DEGs expression in GSE168048 dataset

| Group | AGFG1 | ANXA3 | IL1R1 | RALGAPA2 | MMP8 | WDFY3 | RETN | ORM1 | LRG1 | KLHL2 | PDK4 | LTF | MCTP2 |
| --- | --- | --- | --- | --- | --- | --- | --- | --- | --- | --- | --- | --- | --- |
| Survival | 9.33 | 7.19 | 3.73 | 6.01 | 1.65 | 9.75 | 9.81 | 10.54 | 9.56 | 10.28 | 9.39 | 8.16 | 7.92 |
|  | 9.16 | 9.25 | 3.30 | 5.16 | 2.96 | 8.78 | 12.49 | 10.62 | 10.57 | 9.93 | 10.99 | 11.13 | 8.08 |
|  | 10.06 | 10.23 | 4.78 | 6.02 | 5.11 | 9.61 | 12.94 | 10.15 | 11.20 | 10.01 | 10.71 | 11.46 | 7.91 |
|  | 9.98 | 10.52 | 4.78 | 5.94 | 6.14 | 9.73 | 10.31 | 9.77 | 11.58 | 10.20 | 11.11 | 13.26 | 8.24 |
|  | 9.56 | 10.20 | 3.24 | 5.73 | 6.15 | 9.34 | 10.42 | 10.90 | 9.07 | 9.66 | 8.59 | 13.98 | 7.87 |
|  | 9.55 | 9.04 | 3.72 | 5.92 | 1.69 | 9.75 | 9.85 | 7.68 | 11.09 | 9.90 | 12.17 | 9.51 | 7.76 |
|  | 9.67 | 10.41 | 3.34 | 5.95 | 5.56 | 9.62 | 10.79 | 11.68 | 9.16 | 9.82 | 9.74 | 12.86 | 7.81 |
|  | 9.97 | 10.46 | 3.31 | 6.08 | 3.70 | 9.74 | 10.83 | 9.92 | 10.69 | 9.80 | 9.70 | 11.66 | 7.95 |
| Dead | 10.74 | 12.73 | 5.79 | 7.23 | 7.35 | 10.50 | 13.85 | 12.04 | 11.39 | 10.70 | 10.42 | 13.54 | 9.36 |
|  | 10.94 | 11.52 | 5.01 | 6.97 | 7.13 | 10.44 | 11.68 | 10.57 | 10.87 | 10.51 | 11.64 | 13.31 | 7.45 |
|  | 10.96 | 12.01 | 4.85 | 6.12 | 7.93 | 9.87 | 11.96 | 11.65 | 12.44 | 10.27 | 11.81 | 14.21 | 8.44 |
|  | 11.18 | 12.84 | 5.51 | 6.78 | 9.46 | 10.40 | 13.85 | 12.04 | 12.35 | 10.27 | 11.64 | 15.55 | 9.17 |
|  | 12.09 | 14.28 | 7.95 | 8.14 | 10.16 | 11.64 | 15.64 | 12.54 | 14.28 | 12.12 | 12.59 | 13.29 | 10.92 |
|  | 12.23 | 15.38 | 7.33 | 8.31 | 13.24 | 11.94 | 16.75 | 12.70 | 14.04 | 12.32 | 11.97 | 16.72 | 11.22 |
|  | 11.23 | 12.28 | 5.91 | 6.88 | 8.05 | 10.77 | 12.07 | 12.34 | 12.44 | 11.04 | 13.47 | 13.90 | 8.90 |
|  | 10.46 | 10.39 | 4.62 | 6.46 | 5.40 | 9.99 | 14.28 | 11.26 | 11.18 | 10.43 | 12.27 | 12.26 | 7.97 |

Table S5 logFC values of 12 DEGs in ACLF/NC group or Death/Survival group

| Data | RETN | RALGAPA2 | PDK4 | KLHL2 | ANXA3 | ORM1 | IL1R1 | WDFY3 | LRG1 | LTF | MMP8 | AGFG1 |
| --- | --- | --- | --- | --- | --- | --- | --- | --- | --- | --- | --- | --- |
| GSE142255 (ACLF/NC) | 1.21 | 1.06 | 1.30 | 1.64 | 1.77 | 1.16 | 1.13 | 1.01 | 1.06 | 1.28 | 4.03 | 1.55 |
| GSE168048 (Death/Survival) | 2.83 | 1.26 | 1.67 | 1.01 | 3.02 | 1.73 | 2.10 | 1.06 | 2.01 | 2.60 | 4.47 | 1.57 |

Table S6 Scores and MMP8 expression level in PBMC of 22 HBV-ACLF patients

| **Group**  **Score** | A1 | A2 | A2 | A4 | A5 | A6 | A7 | A8 | A9 | A10 | A11 |
| --- | --- | --- | --- | --- | --- | --- | --- | --- | --- | --- | --- |
| MMP8 | 4.23 | 11.43 | 10.34 | 0.76 | 9.05 | 10.34 | 12.44 | 18.55 | 13.47 | 12.13 | 0.98 |
| MELD | 13.56 | 15.84 | 15.85 | 16.21 | 16.75 | 16.77 | 16.80 | 21.66 | 17.66 | 17.88 | 10.37 |
| COSSH-ACLF II | 5.61 | 5.79 | 6.05 | 5.96 | 6.73 | 6.85 | 5.71 | 7.11 | 6.61 | 6.75 | 3.62 |
| MELD-NA | 18.18 | 18.86 | 21.89 | 20.97 | 18.50 | 23.74 | 17.96 | 24.87 | 18.77 | 23.96 | 11.85 |
| **Group**  **Score** | A12 | A13 | A14 | A15 | A16 | A17 | A18 | A19 | A20 | A21 | A22 |
| MMP8 | 28.32 | 26.37 | 3.75 | 19.21 | 20.61 | 36.14 | 39.87 | 36.53 | 4.04 | 13.01 | 3.71 |
| MELD | 33.21 | 21.63 | 24.81 | 19.84 | 22.85 | 29.31 | 33.00 | 28.22 | 10.63 | 14.38 | 14.06 |
| COSSH-ACLF II | 9.56 | 9.63 | 9.09 | 7.68 | 6.99 | 8.50 | 9.41 | 8.17 | 5.49 | 5.79 | 5.11 |
| MELD-NA | 37.46 | 23.01 | 32.79 | 21.86 | 29.28 | 29.84 | 34.92 | 31.76 | 17.24 | 17.58 | 15.35 |

Table S7 Concentration of M8I determined by preliminary experiments in ACLF mice

| CONC  Lab | 10mg/kg | | | 5mg/kg | | | 2.5mg/kg | | | 2mg/kg | | | 1.5mg/kg | | |
| --- | --- | --- | --- | --- | --- | --- | --- | --- | --- | --- | --- | --- | --- | --- | --- |
| ALT(U/L) | 518 | 876 | 736 | 589 | 523 | 605 | 389 | 301 | 298 | 655 | 609 | 578 | 528 | 487 | 634 |
| AST(U/L) | 702 | 676 | 651 | 415 | 502 | 578 | 341 | 296 | 435 | 482 | 459 | 473 | 765 | 657 | 638 |
| TBil(umol/L) | 45 | 33 | 39 | 33 | 24 | 32 | 15 | 21 | 27 | 43 | 38 | 31 | 33 | 31 | 40 |

Note: CONC, concentration
