# Supplementary material for: MMP8/PPAR-γ regulation of macrophage-mediated inflammatory response in the pathogenesis of acute-on-chronic liver failure
Source: Cell Death Dis. 2026 Apr 27;17(1):556. doi: 10.1038/s41419-026-08793-z (PMC13249895; doi:10.1038/s41419-026-08793-z)
Supplement: Supplementary file 2 — Supplementary Western blots [file 41419_2026_8793_MOESM2_ESM.ppt]

## Slide 1
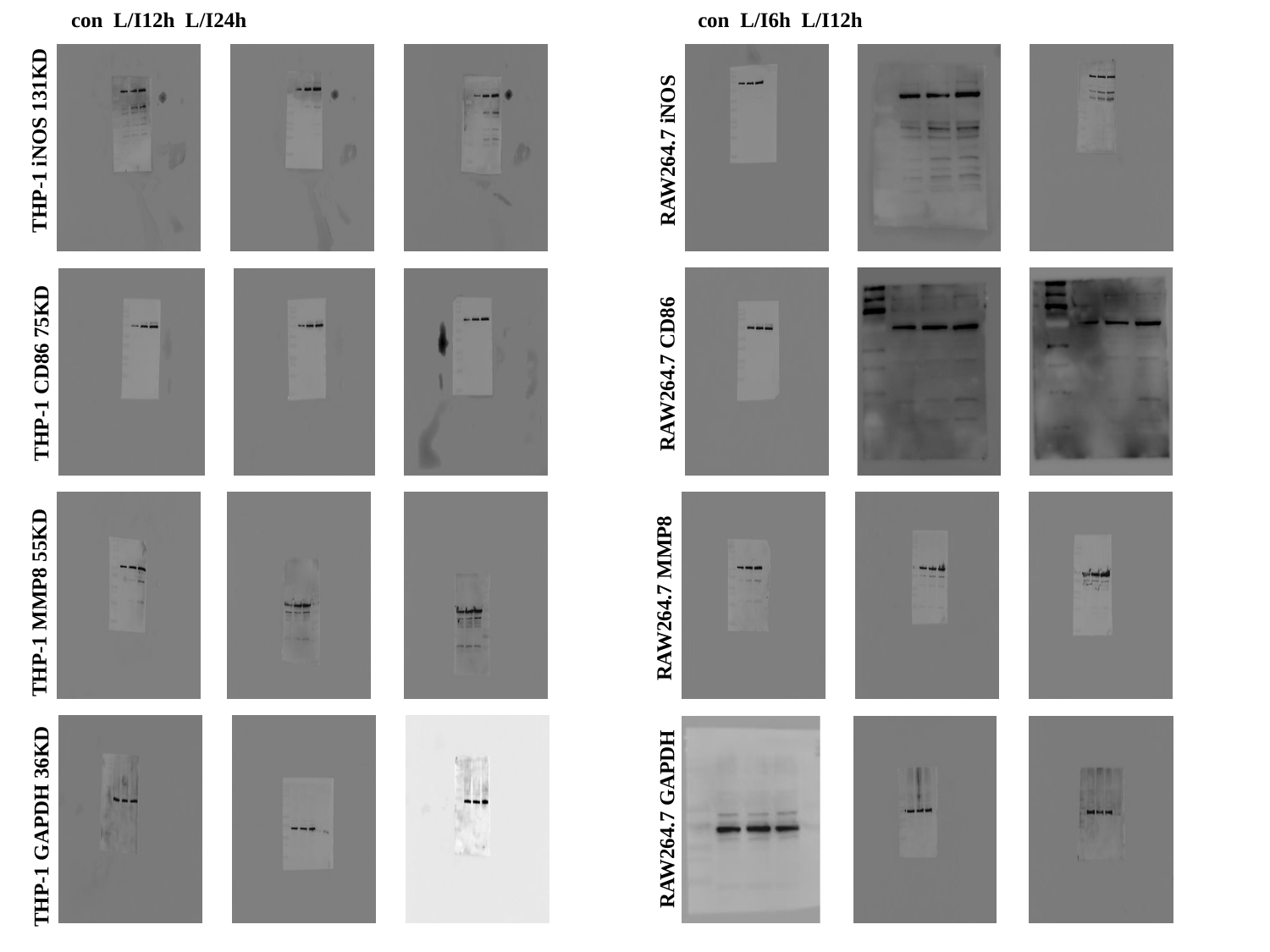

con L/I12h L/I24h
con L/I6h L/I12h
THP-1 iNOS 131KD
RAW264.7 iNOS
THP-1 CD86 75KD
RAW264.7 CD86
THP-1 MMP8 55KD
RAW264.7 MMP8
THP-1 GAPDH 36KD
RAW264.7 GAPDH

## Slide 2
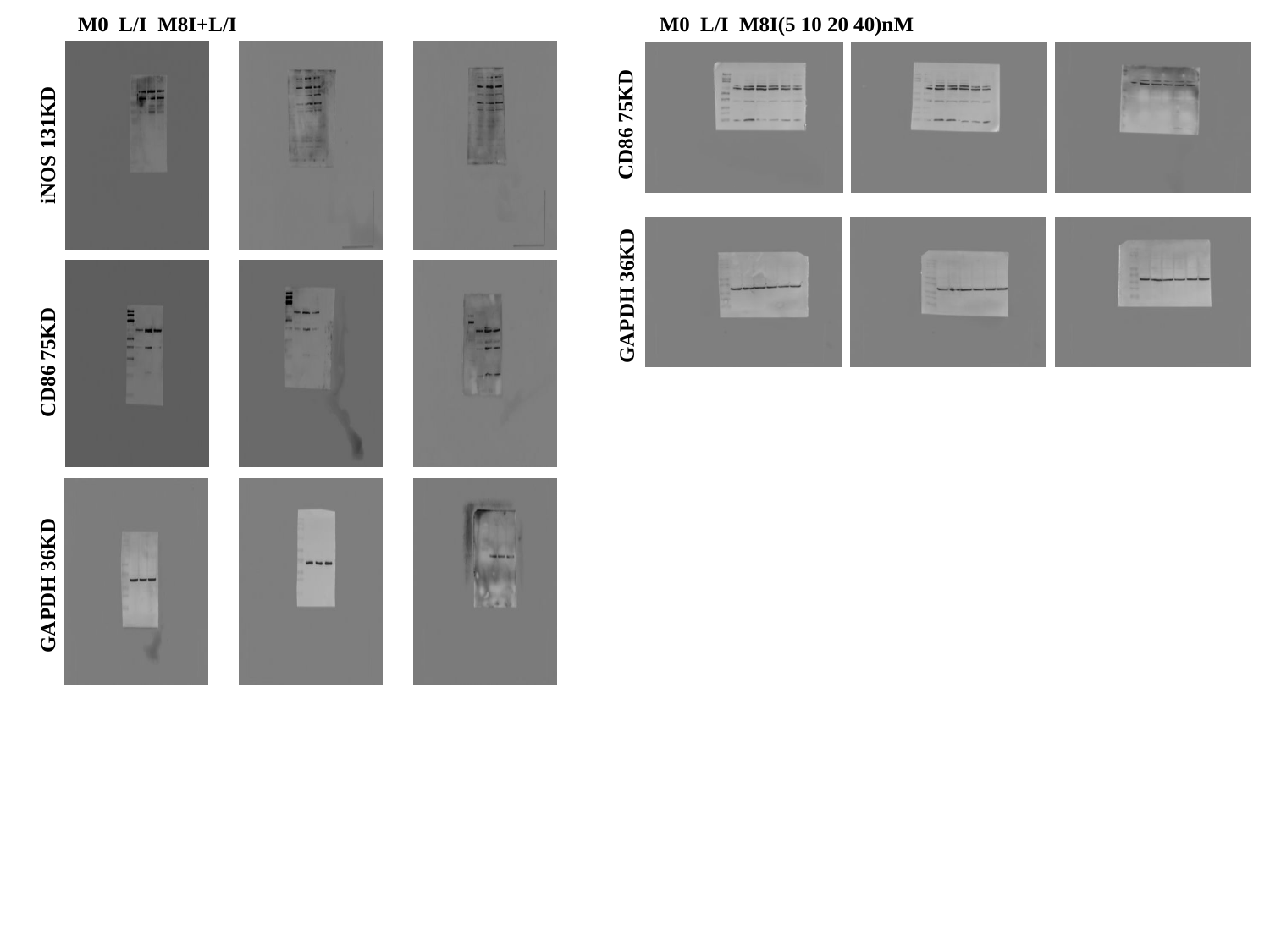

M0 L/I M8I(5 10 20 40)nM
M0 L/I M8I+L/I
CD86 75KD
iNOS 131KD
GAPDH 36KD
CD86 75KD
GAPDH 36KD

## Slide 3
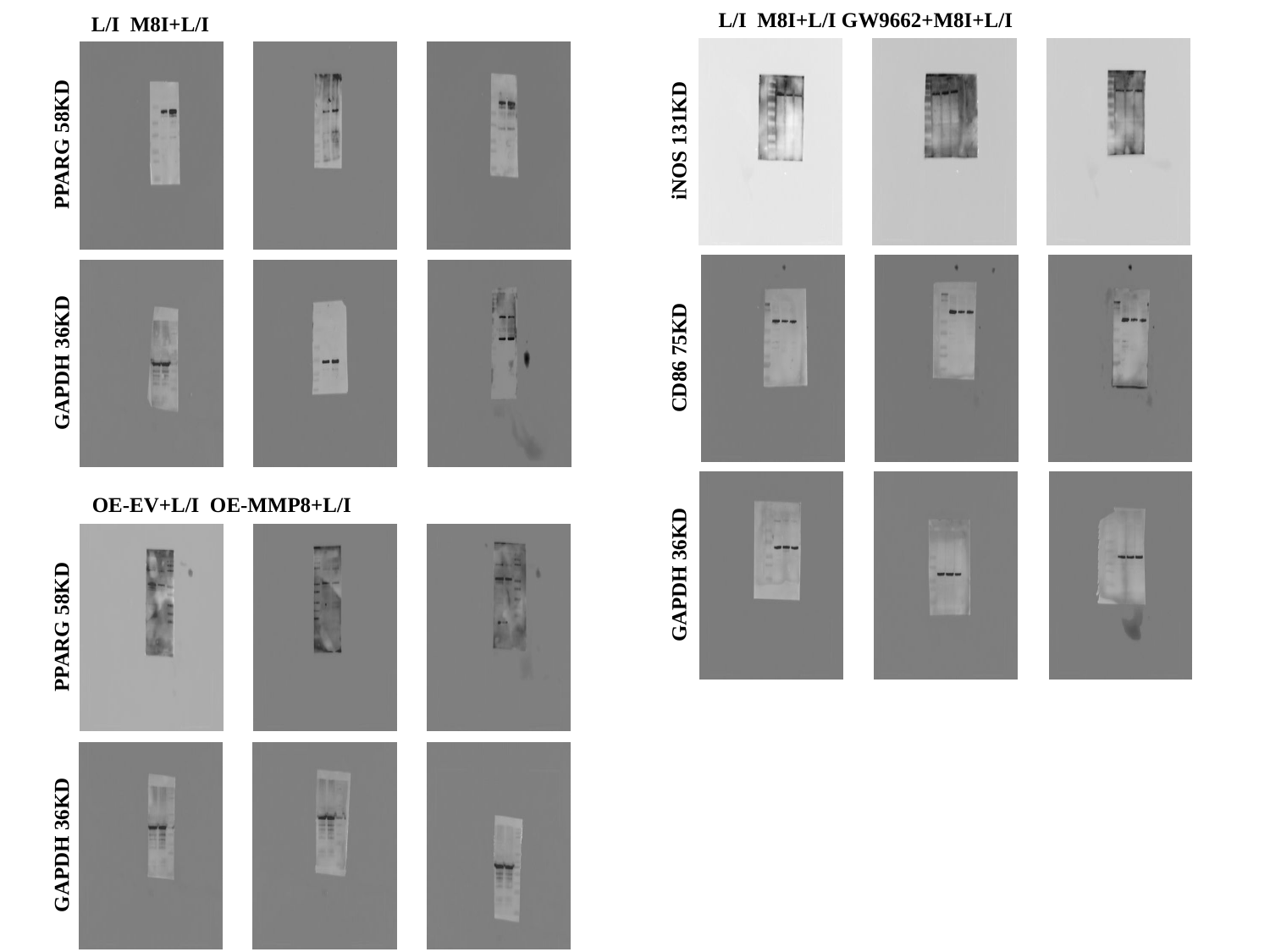

L/I M8I+L/I GW9662+M8I+L/I
iNOS 131KD
CD86 75KD
GAPDH 36KD
L/I M8I+L/I
PPARG 58KD
GAPDH 36KD
OE-EV+L/I OE-MMP8+L/I
PPARG 58KD
GAPDH 36KD

## Slide 4
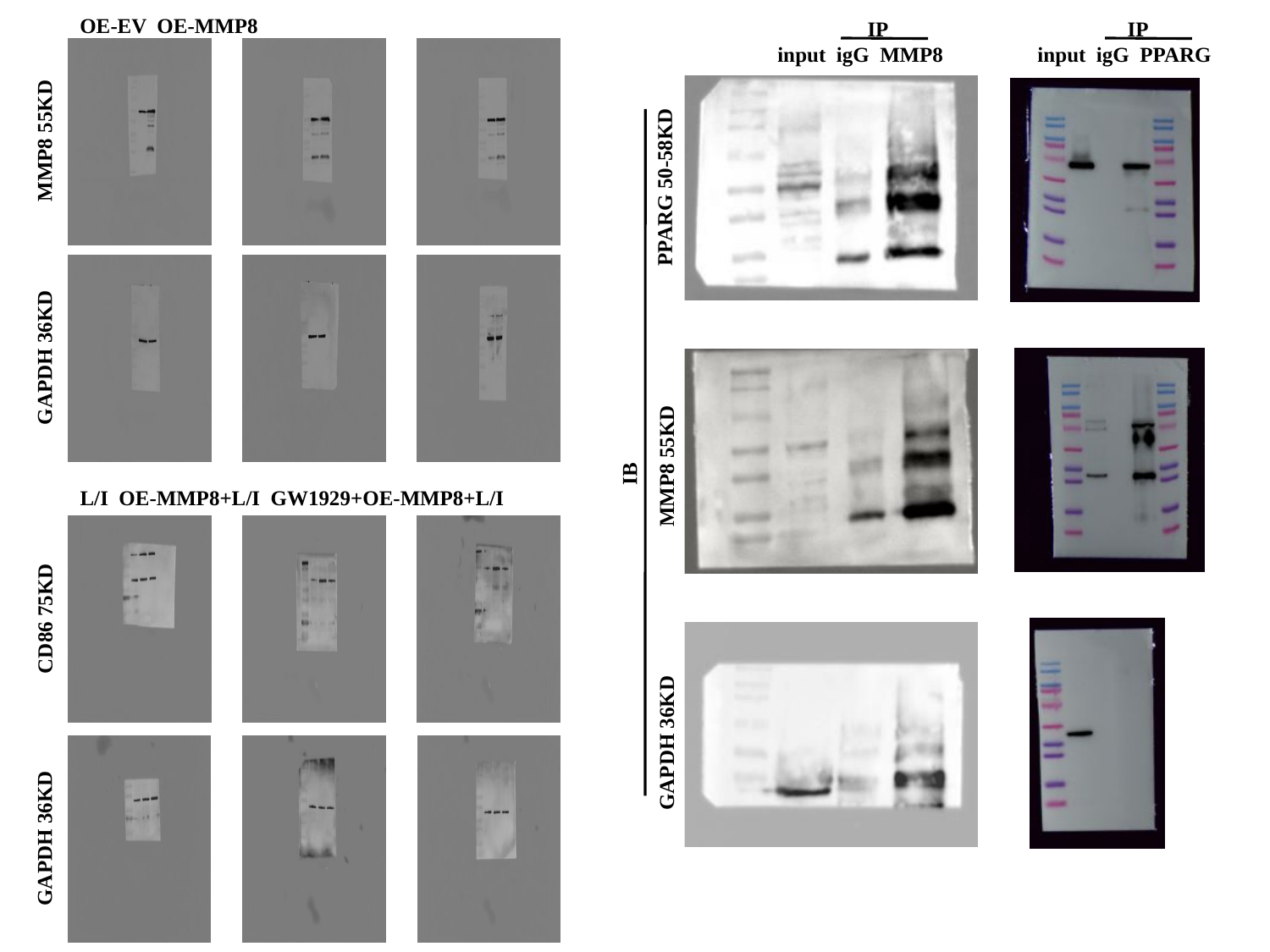

OE-EV OE-MMP8
MMP8 55KD
GAPDH 36KD
 IP
input igG MMP8
 IP
input igG PPARG
PPARG 50-58KD
MMP8 55KD
IB
L/I OE-MMP8+L/I GW1929+OE-MMP8+L/I
CD86 75KD
GAPDH 36KD
GAPDH 36KD

## Slide 5
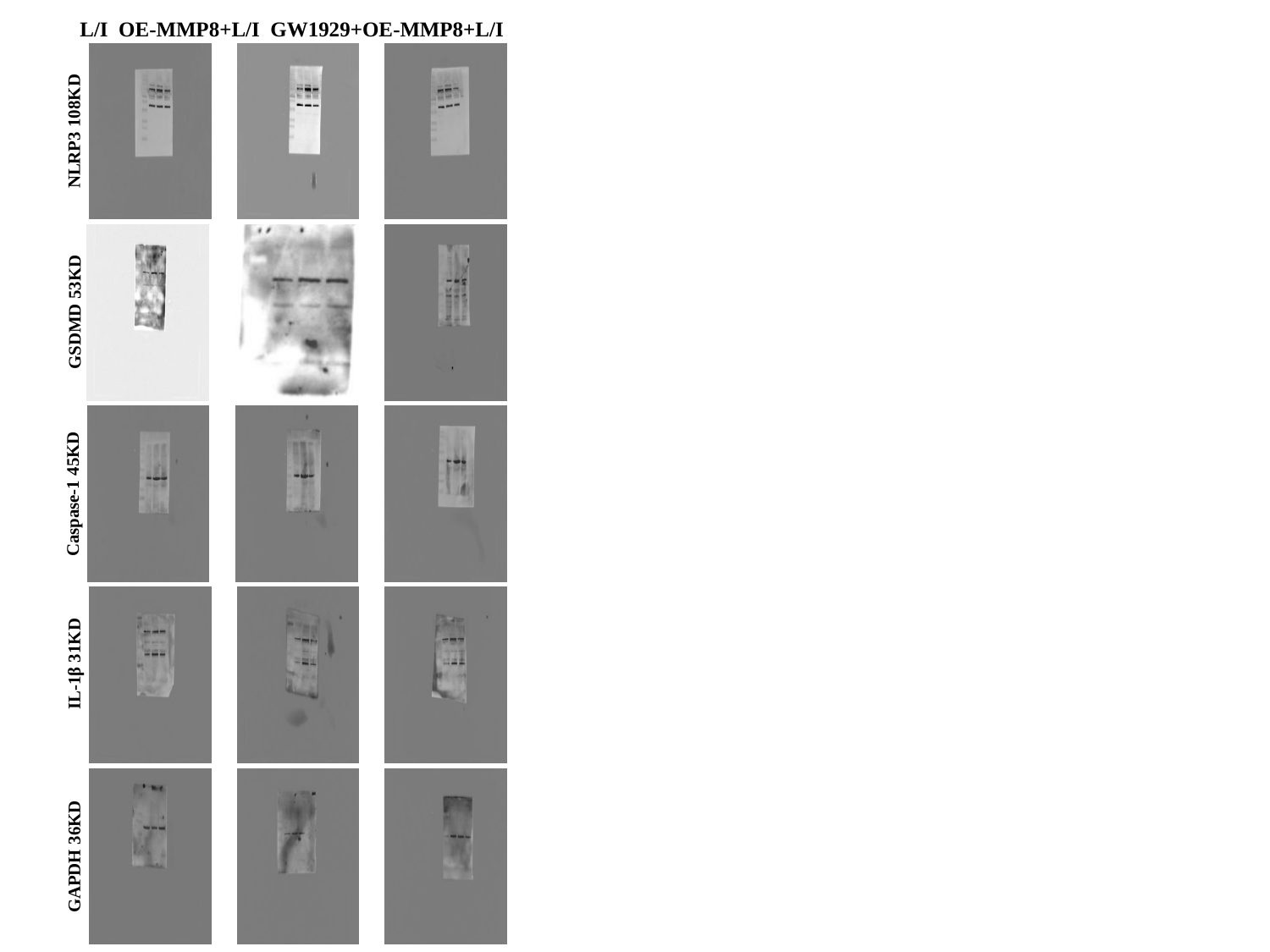

L/I OE-MMP8+L/I GW1929+OE-MMP8+L/I
NLRP3 108KD
GSDMD 53KD
Caspase-1 45KD
IL-1β 31KD
GAPDH 36KD
